# Flavanol Consumption in Healthy Men Preserves Integrity of Immunological‐Endothelial Barrier Cell Functions: Nutri(epi)genomic Analysis

**DOI:** 10.1002/mnfr.202100991

**Published:** 2022-02-16

**Authors:** Dragan Milenkovic, Ana Rodriguez‐Mateos, Margarete Lucosz, Geoffrey Istas, Ken Declerck, Roberto Sansone, René Deenen, Karl Köhrer, Karla Fabiola Corral‐Jara, Joachim Altschmied, Judith Haendeler, Malte Kelm, Wim Vanden Berghe, Christian Heiss

**Affiliations:** ^1^ Department of Nutrition University of California Davis Davis CA 95616 USA; ^2^ INRAE UNH Université Clermont Auvergne Clermont‐Ferrand F‐63000 France; ^3^ Division of Cardiology Pulmonology, and Vascular Medicine Medical Faculty University Hospital Düsseldorf Düsseldorf Germany; ^4^ Department of Nutritional Sciences School of Life Course and Population Sciences Faculty of Life Sciences and Medicine King's College London London UK; ^5^ PPES Department of Biomedical Sciences University of Antwerp (UA) Wilrijk Belgium; ^6^ Biological and Medical Research Center (BMFZ) Heinrich Heine University Düsseldorf Germany; ^7^ Environmentally‐induced Cardiovascular Degeneration Clinical Chemistry and Laboratory Diagnostics Medical Faculty University Hospital and Heinrich‐Heine University Düsseldorf Germany; ^8^ IUF‐Leibniz Research Institute for Environmental Medicine Düsseldorf Germany; ^9^ Clinical Medicine Section Department of Clinical and Experimental Medicine Faculty of Health and Medical Sciences University of Surrey Guildford UK; ^10^ Department of Vascular Medicine Surrey and Sussex NHS Healthcare Trust East Surrey Hospital Redhill UK

**Keywords:** clinical trial, cocoa flavanol, DNA methylation, epicatechin, genomics

## Abstract

**Scope:**

While cocoa flavanol (CF) consumption improves cardiovascular risk biomarkers, molecular mechanisms underlying their protective effects are not understood.

**Objective:**

To investigate nutri(epi)genomic effects of CF and identify regulatory networks potential mediating vascular health benefits.

**Methods and Results:**

Twenty healthy middle‐aged men consume CF (bi‐daily 450 mg) or control drinks for 1 month. Microarray analysis identifies 2235 differentially expressed genes (DEG) involved in processes regulating immune response, cell adhesion, or cytoskeleton organization. Distinct patterns of DEG correlate with CF‐related changes in endothelial function, arterial stiffness, and blood pressure. DEG profile negatively correlates with expression profiles of cardiovascular disease patients. CF modulated DNA methylation profile of genes implicates in cell adhesion, actin cytoskeleton organization, or cell signaling. In silico docking analyses indicate that CF metabolites have the potential of binding to cell signaling proteins and transcription factors. Incubation of plasma obtained after CF consumption decrease monocyte to endothelial adhesion and dose‐dependently increase nitric oxide‐dependent chemotaxis of circulating angiogenic cells further validating the biological functions of CF metabolites.

**Conclusion:**

In healthy humans, CF consumption may mediate vascular protective effects by modulating gene expression and DNA methylation towards a cardiovascular protective effect, in agreement with clinical results, by preserving integrity of immunological‐endothelial barrier functions.

## Graphical abstract

Consumption of cocoa flavanol (CF) by healthy middle‐aged men modulate global gene expression and DNA methylation profile in circulating immune cells. Identified genes are involved in regulation of immune response, cell adhesion, or cytoskeleton organization. These results reveal molecular mechanisms of CF which may mediate vascular protective effects, in agreement with clinical results, by preserving integrity of endothelial barrier functions.

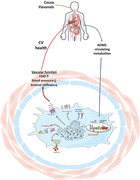

## 1 Introduction

We and others have previously demonstrated in healthy volunteers that the intake of cocoa flavanols (CF) leads to acute and sustained improvements in biomarkers of cardiovascular (CV) function and risk including endothelial function, blood pressure, arterial stiffness, and cholesterol.^[^ ^1^, ^2^ ^]^ The mechanisms of how CF mediates these effects have been investigated in a number of pre‐clinical and in vitro studies,^[^ ^3^, ^4^, ^5^, ^6^ ^]^ but are still not fully understood.^[^ ^7^ ^]^ Taking several limitations of these approaches into account, these studies have indicated that CF could affect vascular cell function^[^ ^8^ ^]^ by interacting with the cellular signaling cascades that regulate transcription factors and as a consequence, expression of genes and proteins.^[^ ^4^, ^9^, ^10^ ^]^ The nutrigenomic impact of dietary flavonoids has not been extensively investigated. In humans, the modulation of gene expression in white blood cells was proposed to be related to the various clinical and biochemical changes that occur during cardiovascular disease (CVD) development.^[^ ^11^ ^]^ Several studies have shown that (poly)phenols can modulate the gene expression profile in white blood cells in healthy volunteers or patients with CVD.^[^ ^12^ ^]^ Long‐term supplementation with monomeric and oligomeric flavanols from grape seeds modulate the expression of genes associated with CVD pathways^[^ ^13^ ^]^ and regular consumption of hesperidin alters leukocyte gene expression to an anti‐inflammatory and anti‐atherogenic profile.^[^ ^14^ ^]^ More recently, nutrigenomic expression analyses described the role of anthocyanins in the regulation of expression of genes and microRNAs related to cell adhesion, migration, immune response, and cell differentiation.^[^ ^15^ ^]^ Systems biology based network analysis revealed that these genes and microRNAs mediate endothelial permeability and monocyte adhesion illustrating complex and multimodal mechanisms of action.^[^ ^16^ ^]^

Gene expression can also be regulated through other modes of action, such as epigenetics.^[^ ^17^ ^]^ DNA methylation is a main epigenetic process which regulates gene and microRNA transcription. Alterations in DNA methylation have been reported to be involved in the development of several human diseases, including cardiovascular diseases.^[^ ^18^, ^19^ ^]^ The ability of (poly)phenols to induce epigenetic changes has been recently highlighted^[^ ^20^ ^]^ including DNA methylation of peripheral leukocytes in humans.^[^ ^21^, ^22^ ^]^ We have also shown that epicatechin metabolites at a physiologically‐relevant concentration can change DNA methylation profile in endothelial cells, changes that have been associated with preservation of endothelial cell functions (prevention of increase in monocyte adhesion and endothelial transmigration) in inflammatory conditions.^[^ ^4^, ^16^ ^]^ Most of these studies evaluated only one type of modification, DNA methylation or gene expression, with hardly any study aiming to perform simultaneous multigenomic analysis. Taken together, (poly)phenols exert their health properties through multigenomic mode of action, mechanisms that are still largely unknown, especially in humans.

Thus, the aim of this study was to identify and characterize the nutri(epi)genomic effects of CF consumption in healthy volunteers by evaluating changes in gene expression and DNA methylation profiles in blood immune cells, evaluating correlations with changes in clinical vascular function biomarkers and cardiovascular‐related gene expression changes, together with functional validation studies in vitro and in silico docking analyses.

## 2 Experimental Section

The ethics committee of the Heinrich‐Heine‐University approved the study protocol, and all subjects gave written informed consent. The study design was described in detail in the previous publication.^[^ ^2^ ^]^ Briefly, the study had performed a double blind randomized parallel group human intervention study in which 100 healthy middle‐aged Europeans completed the study, of which 50 were males and 50 were females. The subgroup analyzed in this current exploratory analysis were all males to avoid the influence of the estrous cycle. The volunteers were given twice daily either placebo control or CF (2 × 450 mg; 900 mg total daily) for 1 month (**Table** **1**). The primary outcome was endothelial function, measured as FMD of the brachial artery. Secondary outcome measures included blood pressure, pulse wave velocity, aortic augmentation index, total, HDL and LDL cholesterol, and Framingham Risk Score. Tertiary endpoints included plasma concentrations of epicatechin metabolites. The methods and results from primary, secondary, and tertiary endpoints were described in the original manuscript. Here, the study presented the analysis of changes in gene expression and epigenetics in blood samples drawn in this study.^[^ ^2^ ^]^ For the nutrigenomic approach blood samples were collected in blood‐stabilizing reagent PAXgene at baseline (day 0), and at day 30 after an overnight fasting period (CF, *n* = 10 and control, *n* = 10).

**Table 1:** Composition of cocoa flavanol and control intervention that were consumed bi‐daily by study participants

	Flavanol	Control
Total cocoa flavanols [mg]	450	ND
Monomers [mg]	73	ND
(−)‐Epicatechin [mg]	64	ND
(−)‐Catechin [mg]	7	ND
(+)‐Catechin [mg]	2	ND
(+)‐Epicatechin [mg]	ND	ND
Dimers‐decamers [mg]	377	ND
Theobromine [mg]	44	46
Caffeine [mg]	10	6
Fat [g]	0	0
Carbohydrates [g]	6	6
Protein [g]	0.1	0.1
Energy [kcal]	25	25
Sodium [mg]	3	3
Potassium [mg]	95	85

ND indicates not detectable.

### 2.1 Cocoa Flavanol (CF)‐Containing Test Drinks

Both interventions, control and CF, were low‐energy fruit‐flavored beverage mix (provided by Mars Inc.), which was standardized and matched in composition. A high‐flavanol cocoa extract (Cocoapro‐processed cocoa extract; Mars Inc.) was the source of flavanols in the CF‐containing drink. The CF‐containing drink provided 450 mg of total CF per serving. The total amount of CF in mg represented the sum of all monomeric flavanols and their oligomers (i.e., procyanidins) with a degree of polymerization up to and including 10 (i.e., DP 1–10). The predominant monomeric flavanol in this drink was (−)‐epicatechin (see Table 1). The control beverage mix did not contain any cocoa extract, and thus it provided 0 mg of CF (control). Given the natural presence of theobromine and caffeine in cocoa extract, both theobromine and caffeine were added to the control beverage mix in order to match the composition of alkaloids in the CF‐containing test product. Coloring was also added so that the 0 mg CF control drink was also indistinguishable in appearance. Compositional details for the 0 mg control and 450 mg CF test drinks were provided in Table 1.

### 2.2 Sample Collection and RNA Preparation

For the nutrigenomic study, blood (2.5 mL) was collected into the PAXgene Blood RNA Tube (PreAnalytix GmbH, Hombrechtikon, Switzerland) by standard venipuncture technique using a blood collection set and a holder. Immediately after blood collection the tubes were inverted 8–10 times and stored upright at room temperature (18– °C) for a minimum of 2 h before freezing at −20 °C for 24 h. For longer storage tubes were transferred to −80 °C. Before starting with the RNA isolation the tubes were thaw at room temperature (18–25 °C) for approximately 2 h and carefully inverted 10 times. RNA was isolated using the PAXgene Blood RNA Kit (PreAnalytix GmbH, Hombrechtikon, Switzerland) as recommended by the manufacturer. The obtained pellet was incubated with proteinase K to degrade proteins. Centrifugation through a PAXgene shredder spin column was carried out for homogenization and removal of cell debris. The lysate was applied to a PAXgene RNA spin column and RNA was bound to the PAXgene silica membrane. After several washing steps and treatment with DNase I to remove bound DNA, RNA was eluted in elution buffer and heat‐denatured.

The quality of obtained total RNA was verified by the Agilent 2100 Bioanalyzer (Agilent, Santa Clara, USA). All samples showed common high‐quality RNA integrity numbers (RINs) between 7.8 and 10. RNA was quantified by photometric Nanodrop measurement. Using this approach, total RNA from 20 samples were obtained (10 volunteers in the control group and 10 volunteers in the CF group).

### 2.3 Microarray Hybridization and Analysis

Synthesis of cDNA and subsequent biotin labeling of cRNA was performed according to the manufacturers´ protocol (3´ IVT Express Kit; Affymetrix, Inc.). Briefly, 100 ng of total RNA was converted to cDNA, followed by in vitro transcription and biotin labeling of caRNA. After fragmentation labeled cRNA was hybridized to Affymetrix PrimeView Human Gene Expression Microarrays for 16 h at 45 °C, stained by strepatavidin/phycoerythrin conjugate and scanned as described in the manufacturers´ protocol.

Data analyses on Affymetrix CEL files were conducted with GeneSpring GX software (Vers. 12.5; Agilent Technologies). The quantile normalization of probe level signal intensities across all samples was used. Input data pre‐processing was concluded by baseline transformation to the median of all samples. The probe had to be expressed above background (i.e., fluorescence signal of a probe set was detected within the 20th and 100th percentiles of the raw signal distribution of a given array) in all replicates in at least one of two conditions to be further analyzed. Differential gene expression was statistically determined by paired *t*‐test comparing replicated treated samples (flavanol or placebo, respectively) to the corresponding individual baseline arrays. The significance threshold was set to *p* = 0.01.

### 2.4 DNA‐Methylation Arrays

gDNA from blood cells was extracted by a DNeasy kit (Qiagen, Courtaboeuf, France). DNA purity and concentrations were determined by UV–vis spectrophotometry (NanoDrop, Thermo Fisher Scientific Inc, Wilmington, DE, USA) and stored at −80 °C until further use. Bisulphite converted DNA from blood leukocytes was hybridized to the Illumina HumanMethylation450 BeadChip arrays (Illumina, San Diego, CA, USA). For each sample, 1 µg of genomic DNA was bisulfite‐converted using an EZ DNA methylation Kit (ZYMO research, Irvine, CA, USA) according to the manufacturer's recommendations. Converted genomic DNA was eluted in 22 µL of elution buffer. DNA methylation level was measured using the Illumina Infinium HD Methylation Assay (Illumina, San Diego, CA, USA) according to the manufacturer's instructions. Briefly, 4 µg of bisulfite‐converted DNA was isothermally amplified overnight (20–24 h) and fragmented enzymatically. Precipitated DNA was resuspended in a hybridization buffer and dispensed onto the Infinium HumanMethylation450 BeadChips (12 samples/chip) using a Freedom EVO robot (Tecan, Männedorf, Switzerland). The hybridization procedure was performed at 48 °C overnight (16–20 h) using an Illumina Hybridization oven. After hybridization, free DNA was washed away and the BeadChips were processed through a single nucleotide extension followed by immunohistochemistry staining using a Freedom EVO robot. Finally, the BeadChips were imaged using an Illumina iScan (Illumina, San Diego, CA, USA). Detection *p*‐values were calculated to identify failed probes as per Illumina's recommendations. No arrays exceeded the quality threshold of >5% failed probes. Filtering of bad quality probes and normalization of raw methylation beta values was conducted using RnBeads package in R.^[^ ^23^ ^]^ Probes with detection *p*‐values higher than 0.01, overlapping with SNPs at the last three bases in its sequence or containing missing values were excluded. Beta mixture quantile dilation (BMIQ) was used to normalize between the two different probe designs (Infinium I and Infinium II).^[^ ^24^ ^]^ Differentially methylated probes were identified using limma R package. Probes with a Benjamini‐Hochberg adjusted *p*‐value below 0.1 and a methylation difference of at least 5% were defined as differentially methylated probes (DMPs). Probes were assigned to genes, CpG island annotations (CpG island, CGI shores, CGI shelves, and open sea) and gene regions (TSS1500, TSS200, 5′UTR, 1st exon, gene body, and 3′UTR) based on the HumanMethylation450 v1.2 Manifest file from Illumina (R package version 1.0.2).

### 2.5 Bioinformatic Analysis

The heat map of gene expression profiles was done using Clustvis tool (https://biit.cs.ut.ee/clustvis/).^[^ ^25^ ^]^ The PCA was constructed using MetaboAnalyst tool (https://www.metaboanalyst.ca).^[^ ^26^ ^]^ The parameters were: rows were centered, and unit variance scaling was applied to rows; rows were clustered using Euclidean distance and average linkage, columns were clustered using maximum distance and average linkage. Gene ontology (GO) annotations of biological processes for differentially expressed genes were conducted using Metacore software (https://portal.genego.com). The GO terms were then classed together using Revigo (http://revigo.irb.hr) online tool and organized in a treemap. To extract maximum biological information of differentially expressed genes, together with gene ontology, genes were also classified into gene networks but also according to their role(s) in cellular or metabolic pathways using Metacore software as well as using online tool Genetrial to place the genes into pathways of Kyoto Encyclopedia of Genes and Genomes (KEGG) and BioCarta database (https://genetrail2.bioinf.uni‐sb.de/) database. Interactions and network of functional groups were searched using Metascape tool (http://metascape.org). Potential transcription factors involved in regulation of gene expression were searched using Metacore software. Venn diagrams were performed using BioVenn (http://www.cmbi.ru.nl/cdd/biovenn/) and Venny (http://bioinfogp.cnb.csic.es/tools/venny/index.html). Identification and 3D visualization of relationships between differentially expressed genes and potential transcription factors and miRNAs was done using OmicsNet tool of MetaboAnalyst tool (https://www.omicsnet.ca). In silico docking approach was used to identify potential interactions between major epicatechin metabolites and transcription factors identified using bioinformatic analysis. Mcule 1‐Click Docking was used for in‐silico docking (https://mcule.com/apps/1‐click‐docking/). Comparative toxicogenomics database (http://ctdbase.org) was used to search for gene‐disease associations. To search for gene‐COVID19 associated genes using gene expression data deposited in the GEO, Enrichr tool (https://maayanlab.cloud/Enrichr/) was used. Networks of pathways, their interactions and genes involved with each pathway were also searched using GluGO Cytoscape application which allows to create and visualize functionally grouped networks.^[^ ^27^ ^]^ Cytoscape version 3.7.2 was used for the analysis.^[^ ^28^ ^]^

### 2.6 Correlation Analyses

This study performed the correlation analysis between identified changes in expression of the genes affected following 4‐week CF consumption with changes in clinical parameters/vascular function biomarkers including FMD, office and central systolic and diastolic blood pressure, PWV. The study used the Spearman correlation and the pairs correlated were chosen using a correlation coefficient greater than abs (0.80) and a *p*‐value <0.05. The analysis was done using the Hmisc package (https://github.com/harrelfe/Hmisc) in R. The Lares package (https://github.com/laresbernardo/lares) was used to visualize the top 110 variables that were correlated with FMD, SBP, DBP, and PWV.

The study then performed prediction of disease traits by performing correlation analysis between changes in the expression of genes following 4‐week CF consumption and those observed in patients with coronary artery disease extracted from the gene expression profile from Grayson et al.^[^ ^29^ ^]^ study which were deposited in GEO (gene expression omnibus) database under ID number: GSE23561. The study was performed to obtain gene expression profiles in peripheral blood from patients with coronary artery disease, type 2 diabetes and their precursor state, metabolic syndrome to those of control subjects, and subjects with rheumatoid arthritis. The study used Human 50K Exonic Evidence‐Based Oligonucleotide array. The identification of differentially expressed genes between patients with coronary artery disease and healthy patients was performed using GEO2R (http:// www.ncbi.nlm.nih.gov/geo/info/geo2r.html). GEO2R was a NCBI web tool to compare two or more groups of samples in GEO series for identifying differentially expressed genes across experimental conditions. The differentially expressed genes were screened according to *p* values < 0.05. Pearson's correlation analysis between genes identified as differentially expressed following CF consumption and patients with coronary artery disease was performed using online Wessa statistical software (www.wessa.net/rwasp_ correlation.wasp/).

### 2.7 In vitro Validation of Cellular Effects

#### 2.7.1 Cell Adhesion Assay

Primary human umbilical vein endothelial cells (HUVECs) (Lonza, Walkersville, MD, USA) were used at passage 3 and were cultured in a phenol red‐free endothelial growth medium (EGM) supplemented with 2% fetal bovine serum (FBS), 0.4% fibroblast growth factor, 0.1% vascular endothelial growth factor, 0.1% heparin, 0.1% insulin‐like growth factor, 0.1% ascorbic acid, 0.1% epidermal growth factor, and 0.04% hydrocortisone (all from Lonza). Experiments were performed in 24 well plates (Becton Dickinson, Le Pont de Claix‐Cedex, France). A human monocytic cell line (THP1) (ATCC, Manassas, VA, USA) was cultured in the RPMI medium (Pan Biotech) supplemented with 2% FBS (Sigma, Saint Quentin Fallavier, France). Both cultures were maintained at 37 °C and 5% CO_2_.

HUVECs were allowed to proliferate until they reached 80% of confluence and the monolayer was stimulated for 4 h with TNFα at 0.1 ng mL^−1^ or PBS/BSA (0.01‰, negative control). Following TNFα stimulation, 50 µL of a 5 × 10^6^ mL^−1^ THP1 cell suspension were exposed for 2 h to CF metabolites isolated from plasma, using solid‐phase extraction method,^[^ ^30^ ^]^ of volunteers that participated in the cocoa‐flavanol intervention study. The isolated metabolites were resuspended in the same volume of culture medium as volume used for solid‐phase extraction in order to have the same concentration in the culture medium as in plasma. The cells were then added to each well and cells were further incubated for 1 h. Non‐adhering THP1 cells were rinsed away using PBS 1× and the wells were fixed with crystal violet 0.5% in ethanol (Sigma). The number of attached monocytes was counted for each well in three random microscopic fields defined by an eyepiece. Triplicates for each condition were performed in three independent experiments.

#### 2.7.2 Circulating Angiogenic Cells

**Figure 1:**
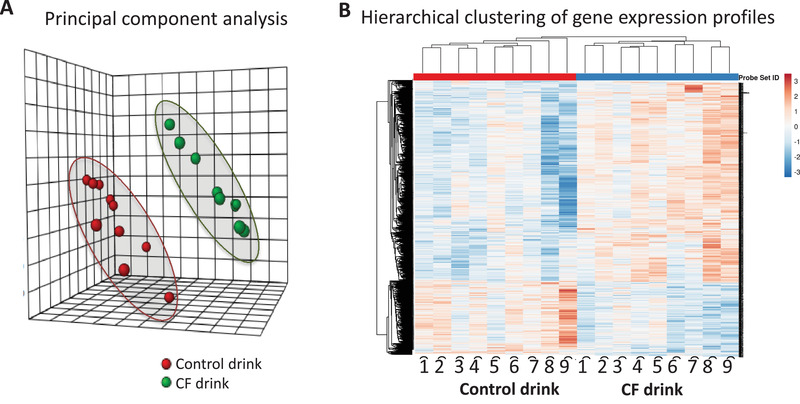
Comparison of gene expression profiles in immune cells from volunteers that consumed 30 days cocoa flavanol (CF) drinks and volunteers control drinks using A) principal component analysis (red; control drink, green: flavanol‐rich drink) and B) heatmap analysis of gene expression as assessed before and after consumption.

CACs were differentiated ex vivo from peripheral blood mononuclear cells (PBMNC) as previously described.^[^ ^31^ ^]^ Briefly, blood was drawn from the cubital vein into vacuum tubes pre‐filled with a liquid density gradient medium and PBMNCs were isolated based on the Ficoll method (Vacutainer CPT, Becton Dickinson, Franklin Lakes, NJ). In order to remove mature endothelial cells from the harvested cell population, the cells were preplated on fibronectin‐coated culture plates for 1 day in EBM‐2 MV (supplemented with Singlequots, 20% fetal bovine serum, HyClone, Logan, UT). The initially firmly adherent cells were discarded and the non‐adherent cells (>95%) were moved to a new dish and cultured for another 6 days, during which time many cells (10% on average) became newly adherent. Cell migration was quantified by a transwell chemotaxis assay using a modified Boyden chamber.^[^ ^31^ ^]^ Migration of CACs was measured as follows: Cells (2 × 10^4^) were plated in the upper of two chambers divided by a membrane with 8 µm pores (Corning Transwell). The study tested the effect of CF metabolites on chemotactic properties of cells toward a gradient of vascular endothelial growth factor (VEGF, Sigma) which was only present in the lower chamber (50 ng mL^−1^). The suspension medium of cells and medium for bottom chamber was supplemented with 1%, 5%, or 10% plasma pooled from four healthy subjects of the study drawn before at 0 h or at 2 h after 450 mg CF. The concentrations of CF metabolites in 0 h plasma was below the limit of detection and profile of 2 h plasma was shown in **Figure** **1**B. The metabolites were identified and quantified as described earlier.^[^ ^2^ ^]^ In parallel, experiments to test the role of eNOS on chemotaxis were conducted by adding the NOS inhibitor L‐NMMA (100 µmol L^−1^) to both the upper and lower chamber. The number of migrated cells was determined after 6 h on five random 100× optical fields per membrane.

## 3 Results

### 3.1 Baseline Characteristics of the Subpopulation

Information on volunteer enrollment, randomization, inclusion and exclusion, as well as reasons for the latter are detailed in Sansone et al.^[^ ^2^ ^]^ The baseline characteristics of the volunteers with available blood samples for microarray analysis are presented in **Table** **2**. The clinical baseline characteristics (body mass index, blood pressure, total cholesterol, LDL, HDL, and fasting glucose) were within normal limits.

**Table 2:** Baseline characteristics of study population

n		20	
Age [y]	43.3	±	8.7
Male (n)		20	
Body mass index [kg m^−2^]	25.0	±	1.7
Height [m]	1.80	±	0.07
Weight [kg]	80	±	10
Creatinine [mg dL^−1^]	0.9	±	0.2
Total cholesterol [mg dL^−1^]	204	±	30
LDL cholesterol [mg dL^−1^]	140	±	30
HDL cholesterol [mg dL^−1^]	52	±	10
Triglycerides [mg dLl^−1^]	100	±	39
Fasting plasma glucose [mg dL^−1^]	89	±	10
HbA1c [%]	5.5	±	0.4
Systolic blood pressure [mmHg]	132	±	13
Diastolic blood pressure [mmHg]	81	±	9
Heart rate [min]	64	±	8
C‐reactive protein [mg dL^−1^]	0.1	±	0.03
Hemoglobin [mg dL^−1^]	15.0	±	0.6
Leucocytes [1000 µL^−1^]	5.5	±	0.2

Values are mean and standard deviation.

### 3.2 CF Consumption Modulates Expression of Genes in Humans

**Figure 2:**
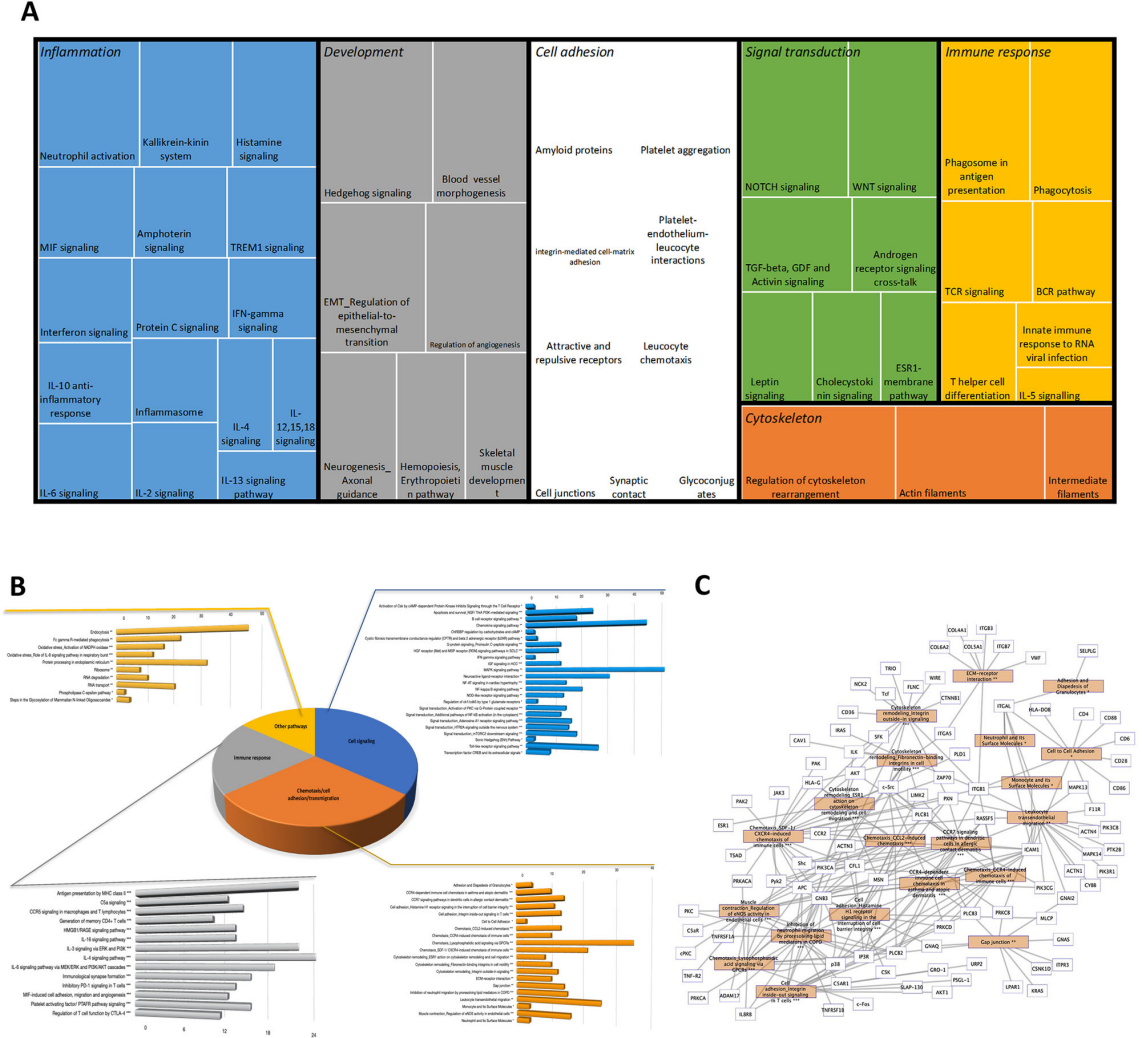
Functional analysis of differentially expressed genes. Treemap of gene networks obtained using a text mining approach A), cellular pathways obtained from BioCarta, KEGG, and Metacore using GeneTrail B), and gene‐pathway interaction network involved in chemotaxis, cell adhesion, and migration C).

Our first analysis was to compare global gene expression profiles obtained for all volunteers using partial least squares discriminant analysis (PLS‐DA). This analysis revealed that the expression profiles of genes of volunteers that had received CF are separated from the gene expression profiles of the volunteers after control treatment and form distant groups (**Figure** **2**A). Hierarchical clustering of gene expression profiles also identified two groups of profiles, corresponding to before and after CF consumption, with opposite gene expression profile (Figure 2B). These analyses suggest that chronic consumption of CF leads to an overall significant change in global gene expression profile.

Following this observation, a statistical analysis of gene expression data was performed on the processed microarray data. This analysis revealed differential expression of 2235 genes following CF consumption when compared to control, of which 1147 were upregulated and 1088 were downregulated (Figure S1, Supporting Information). The fold‐change identified varied from −1.5 for downregulated genes to 2.8 for upregulated genes. This observation indicates that regular consumption of CF by healthy volunteers can significantly impact the expression of genes in blood cells.

### 3.3 CF Modulates Expression of Genes Involved in Inflammation and Cell Adhesion

To identify biological functions of differentially expressed genes in response to CF, bioinformatic analyses were performed using different tools. Enrichment for gene ontology (GO) terms was performed using the Metacore software which classified the differentially expressed genes according to their biological processes. Identified GO terms were then organized according to their relationship and visually represented in a treemap (Figure S2, Supporting Information). We observed that genes which expression modified by 4‐week CF intake are involved in a number of biological processes including response to oxidative stress, cell‐cell adhesion, apoptotic process, or cellular transport.

**Figure 3:**
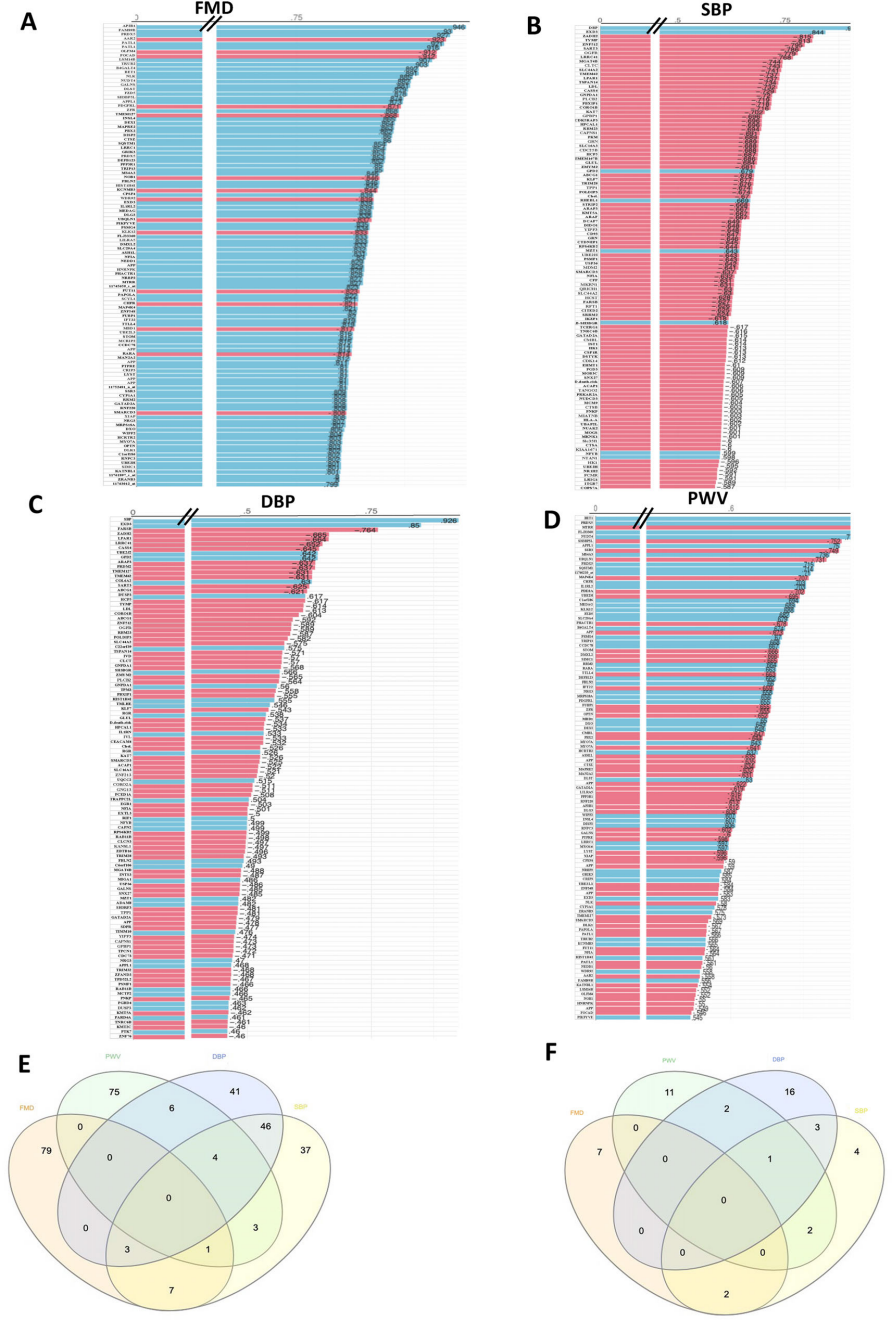
Correlation analysis between identified differentially expressed genes and measured parameters: flow‐mediated dilation (FMD; A); systolic blood pressure (SBP; B); diastolic blood pressure (DBP; C); pulse wave velocity (PWV, D). Venn diagram of the genes identified as associated with the four clinical parameters E) and Venn diagram of the pathways of the genes associated with the four parameters.

The differentially expressed gene products are in close relation with other gene products and form together an entire network that regulates integrity of endothelial barrier cell function. Metacore software was also used to investigate these gene networks and their corresponding biological functions using a text mining approach. Using this approach, we observed that differentially expressed genes form networks involved in immune response and inflammation, cell adhesion, cell cytoskeleton, development, and signal transduction as represented in the treemap in **Figure** **3**A. Different networks were identified within functional groups. Within the immune response/inflammation group, for example, IL‐5, IL‐12, IL15 signaling, or IL‐10 anti‐inflammatory response networks were identified. Within the cell cytoskeleton group, regulation of cytoskeleton rearrangement or actin filaments networks were identified. Within the signal transduction group, TGF or WNT signaling networks were identified. As for GO analysis, this gene network analysis also revealed that differentially expressed genes are involved in different processes, some known to be related to development of cardiometabolic diseases.

Following gene network analyses, we searched for most over‐represented pathways in which identified differentially expressed genes are involved. As shown in Figure 3B, the pathways represented are involved in the regulation of cell signaling, cell adhesion/migration, and immune response, which is in accordance with the analysis obtained by gene network analyses described above. Among the pathways identified are pathways regulating cell adhesion and migration are adhesion and diapedesis of granulocytes, pathways regulating immune cell chemotaxis, leukocyte transendothelial migration, surface molecules of immune cells, cell–cell adhesion or cytoskeleton remodeling in migration. A network analysis of pathways involved in cell chemotaxis, adhesion and migration together with differentially expressed genes showed that these genes form complex network of interactions regulating this important cellular function. This pathway analysis suggests that CF induced gene expression changes modulate immune cell interaction with endothelial cells, their chemotaxis to vasculature and their transmigration into vascular wall. The pathway analysis also revealed over‐represented pathways regulating interleukins signaling pathways, such as IL‐4, IL‐16, IL‐6, or IL‐3 signaling pathways, PD‐1 signaling in T cells or inflammatory platelet‐activating factor receptor signaling. This observation suggests that CF could also affect inflammatory properties of blood cells by modulating the expression of genes encoding pro‐ or anti‐inflammatory molecules. Differentially expressed genes are also implicated in several cell signaling pathways, such as toll‐like receptor signaling pathway, PI3K signaling pathway, MAPK signaling pathway, chemotaxis signaling pathway, or NF‐κB signaling pathway. The bioinformatic analysis also revealed several pathways involved in the regulation of other cellular functions, such as regulation of oxidative stress, endocytosis, respiratory burst, or protein processing in the endoplasmic reticulum. An interactive network between identified pathways and differentially expressed genes has been obtained showing interactions between genes modulated by CF consumption and associated pathways (Figure 3C, Figure S3, Supporting Information). Taken together, CF consumption induced gene expression changes that preserve integrity of immunological‐endothelial barrier cell processes.

Upon searching for protein interaction networks of differentially expressed genes via the STRING algorithm (https://string‐db.org/), an interactome of over 3200 interactions was built (Figure S4A, Supporting Information). Key node proteins having the highest number of interactions were searched and 27 “hubs” interacting with 15–39 proteins, were identified (Figure S4B, Supporting Information). Pathway analyses of these key node genes showed that they are involved in pathways regulating cell adhesion, protein processing in endoplasmic reticulum, cytoskeleton organization, focal adhesion, or leukocyte transendothelial migration. In line with pathway enrichment analysis, protein network analysis reveals similar cellular biological functions related to adhesion of immune cells to and transmigration through the vascular wall.

Following these analyses, we used the comparative toxicogenomics database (CTD) to analyze the associations between genes and human diseases by comparing of previously identified gene‐disease associations and online gene expression data. We observed high degree of similarity between genes which expression has been modulated by CF and genes identified in development of cardiovascular diseases, including vascular diseases, heart diseases, cerebrovascular disease, and hypertension (**Table** **3**).

**Table 3:** Enriched diseases with number of inferred gene‐diseases relationship identified using gene set analysis (from The Comparative Toxicogenomics Database http://ctdbase.org)

Disease name	Disease ID	*p*‐value	Corrected *p*‐value	Annotated genes number
Cardiovascular diseases	MESH:D002318	8.61E‐47	1.80E‐43	212
Heart diseases	MESH:D006331	1.61E‐39	3.37E‐36	165
Vascular diseases	MESH:D014652	7.01E‐25	1.46E‐21	124
Myocardial ischemia	MESH:D017202	1.64E‐16	3.43E‐13	64
Cerebrovascular disorders	MESH:D002561	5.72E‐11	1.19E‐07	37
Brain ischemia	MESH:D002545	5.49E‐10	1.15E‐06	24
Cardiovascular abnormalities	MESH:D018376	2.65E‐08	5.53E‐05	32
Heart defects, congenital	MESH:D006330	3.91E‐08	8.16E‐05	30
Arrhythmias, cardiac	MESH:D001145	8.79E‐08	1.83E‐04	33
Atrial fibrillation	MESH:D001281	3.76E‐07	7.85E‐04	25
Hypertension	MESH:D006973	4.70E‐07	9.80E‐04	28
Cardiomyopathies	MESH:D009202	2.51E‐06	0.00524	33
Heart failure	MESH:D006333	3.53E‐06	0.00738	22
Stroke	MESH:D020521	3.89E‐06	0.00813	17

### 3.4 Changes in Gene Expression Correlates with Vascular Effect of CF

**Figure 4:**
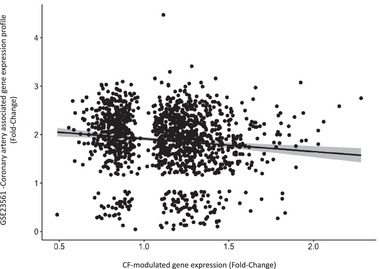
Correlation analysis between genes modulated by CF and gene expression profiles in patients with coronary artery diseases (GSE23561).

Investigation of correlations between genes identified as having changes in expression following consumption of CF and changes in measured clinical parameters identified several dozens of significant correlations (*p < *0.05, **Figure** **4**A–D). A number of genes showed significant correlations, both positive and negative, with FMD, and among genes presenting highest correlation values are *GFI1B* (*r* = 0.94), *CAPNS1* (*r* = 0.93), *GBA* (*r* = 0.93), *SLA2* (*r* = 0.87), *RASGRP1* (*r* = 0.87). Also, a number of gene changes showed significant correlations with PWV, a marker of vascular stiffness and ageing, including *BET1* (*r* = 0.89), *PRDX5* (*r* = 0.84), *MTRR* (*r* = −0.81), and *APPL1* (*r* = 0.75). For blood pressure we identified genes including *FARSB* (*r* = −0.76), *LPAR1* (*r* = −0.66), *TYMP* (*r* = −0.81), *SLC44A2* (*r* = −0.74). Comparison of genes of which changes in the expression were identified as correlated with each of the four clinical parameters, showed little overlap of genes (Figure 4E). The highest number of common genes were identified between SBP and DBP, suggesting potential common mechanisms of action, mechanisms what seem to be different than those regulating FMD and PWV. In the similar manner, little overlap of genes between vascular parameters (FMD and PWV) is suggestive of different mechanisms of regulation involved in their regulation by CF. We than aimed to identify cellular pathways in which these genes are involved in (Figure 4F). We observed that they are regulating processes like PI3K‐Akt signaling pathway, focal adhesion, cell adhesion, or integrin‐mediated cell adhesion. Comparison of pathways obtained using genes correlated for each of the four clinical parameters, have once again revealed few common pathways, with most common pathways being observed once again between DBP and SBP. Taken together, the data indicate that changes in the expression of some of the identified genes were linked with changes in individual vascular parameters, and point to different mechanisms and pleiotropic effects of CF between studied biomarkers.

### 3.5 Genomic Response to CF Is Inversely Correlated with Gene Expression Profiles in Peripheral Blood from Patients with Coronary Artery Disease

**Figure 5:**
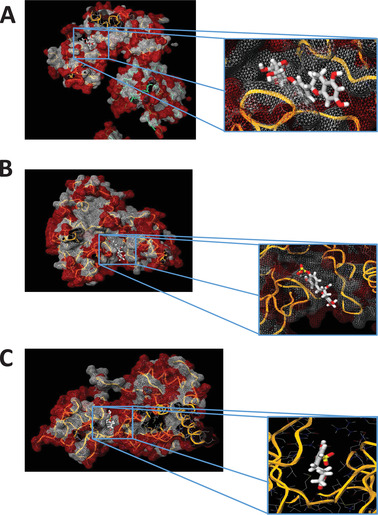
In silico docking with cell signaling proteins involved in the regulation of identified transcription factors. A) RelA/p65 of NF‐κB with EC3’G; B) RelA/p65 of NF‐κB with EC3’S; C) JNK (cell signaling protein involved in the regulation of identified transcription factors) with 5‐(4′‐hydroxyphenyl)‐γ‐valerolactone‐3′‐sulfate (γVL3′S).

Our next aim was to assess whether a 4‐week CF intervention and coronary artery disease associated genomic expression modifications present opposite genomic profiles. We performed Pearson correlation analysis between gene expression changes after CF intervention in the current study^[^ ^32^ ^]^ with gene expression changes identified in patients with coronary artery disease (GSE23561 gene dataset from Gene Expression Omnibus). Interestingly, based on fold‐change in gene expression, a negative correlation (*r* = −0.15; *p* = 7.4 × 10^–8^) was obtained. This observation suggests that CF consumption exerts overall opposite changes in gene expression in comparison to gene expression changes observed in patients with coronary artery disease (**Figure** **5**), highlighting the potential importance of the observed nutrigenomic and clinical data.

### 3.6 Transcriptional and Post Transcriptional Regulators of Gene Expression

Regulation of the expression of identified genes is potentially a consequence of the interaction of CF metabolites with cell signaling proteins and consequently modulation of transcription factors activity. To identify potential transcription factors involved in the nutrigenomic effect observed, transcription factors were searched using the OmicsNet online tool. Among the transcription factors identified were SP1, STAT3, RelA, and NF‐κB (**Table** **4**). This analysis revealed potential transcription factors that may underlie the nutrigenomic effect of chronic CF consumption. The activity of these transcription factors could be regulated by the interaction of CF metabolites with cell signaling receptors and downstream kinases which may change their activity and consequently affect the activity of the transcription factor(s).

**Table 4:** Identification of transcription factors potentially modulated by CF consumption and underlying in the genomic modifications by CF

Symbol	Name	Number of hits
Transcription factor		
SP1	Specificity Protein 1	256
RELA	RELA Proto‐Oncogene/p65	187
NFKB1	Nuclear Factor Kappa B Subunit 1	184
TP53	Tumor protein p53	115
E2F1	E2F Transcription Factor 1	98
STAT3	Signal transducer and activator of transcription 3	96
JUN	Jun Proto‐Oncogene	87
MYC	MYC Proto‐Oncogene	69
HIF1A	Hypoxia Inducible Factor 1 Subunit Alpha	61
YY1	Yin Yang 1	57
SP3	Specificity Protein 3	56
STAT1	Signal transducer and activator of transcription 1	56
AR	Androgen receptor	52
EGR1	Early Growth Response 1	51
HDAC1	Histone Deacetylase 1	51
CREB1	CAMP Responsive Element Binding Protein 1	48
ESR1	Estrogen receptor alpha	45
ETS1	ETS Proto‐Oncogene 1	43
TFAP2A	Transcription Factor AP‐2 Alpha	42
BRCA1	Breast Cancer Type 1 Susceptibility Protein	39
PPARG	Peroxisome proliferator‐activated receptor gamma	39
SIRT1	Regulatory Protein SIR2 Homolog 1	38
WT1	Wilms Tumor 1	38
EP300	E1A Binding Protein P300	35
IRF1	Interferon Regulatory Factor 1	35
SPI1	Spi‐1 Proto‐Oncogene	35
GATA1	GATA Binding Protein 1	33
FOS	Proto‐Oncogene C‐Fos	31
USF1	Upstream Transcription Factor 1	31
CEBPB	CCAAT Enhancer Binding Protein Beta	29

We also performed bioinformatic analyses, using OmicsNet software, to identify potential microRNAs that could be involved in the regulation of the expression of genes identified as differentially expressed following CF consumption. We identified several miRNAs and the top 30 are presented in the **Table** **5**. Among these miRNAs were mir‐16‐5p, mir‐26b‐5p, mir‐335‐5p, mir‐92a‐3p, and let‐7b‐5p. We then analyzed functional integrations of differentially expressed genes and the protein–protein interaction with identified potential transcriptional factors and miRNAs (Figure S5, Supporting Information). This analysis showed a multi‐pharmacological mode of action of CF in blood cells following chronic consumption of CF, cell miRNA and gene expression modifications, involved in regulation of inflammation, cytoskeleton organization, and chemotaxis of immune cells.

**Table 5:** Identification of potential microRNAs involved in the post‐translational regulation of genes using OmicsNet database (https://www.omicsnet.ca)

Symbol	Name	Number of hits
micro RNA		
mir‐16‐5p	microRNA‐16‐5p	667
mir‐26b‐5p	microRNA‐26b‐5p	625
mir‐335‐5p	microRNA‐335‐5p	625
mir‐92a‐3p	microRNA‐92a‐3p	612
let‐7b‐5p	let‐7b‐5p	532
mir‐124‐3p	microRNA‐124‐3p	503
mir‐93‐5p	microRNA‐93‐5p	469
mir‐17‐5p	microRNA‐17‐5p	456
mir‐615‐3p	microRNA‐615‐3p	435
mir‐155‐5p	microRNA‐155‐5p	431
mir‐193b‐3p	microRNA‐193b‐3p	414
mir‐106b‐5p	microRNA‐106b‐5p	408
mir‐20a‐5p	microRNA‐20a‐5p	407
mir‐484	microRNA‐484	385
mir‐1‐3p	microRNA‐1‐3p	376
mir‐218‐5p	microRNA‐218‐5p	349
mir‐15b‐5p	microRNA‐15b‐5p	329
mir‐192‐5p	microRNA‐192‐5p	329
mir‐30a‐5p	microRNA‐30a‐5p	329
mir‐20b‐5p	microRNA‐20b‐5p	326
mir‐186‐5p	microRNA‐186‐5p	325
mir‐1‐1	microRNA‐1‐1	322
mir‐519d‐3p	microRNA‐519d‐3p	320
mir‐24‐3p	microRNA‐24‐3p	319
mir‐15a‐5p	microRNA‐15a‐5p	306
mir‐19b‐3p	microRNA‐19b‐3p	293
mir‐34a‐5p	microRNA‐34a‐5p	289
mir‐98‐5p	microRNA‐98‐5p	278
mir‐21‐5p	microRNA‐21‐5p	276
mir‐195‐5p	microRNA‐195‐5p	272

### 3.7 In silico Docking Analysis Suggest Potential Binding Between Epicatechin Metabolites and Cell Signaling Proteins

**Figure 6:**
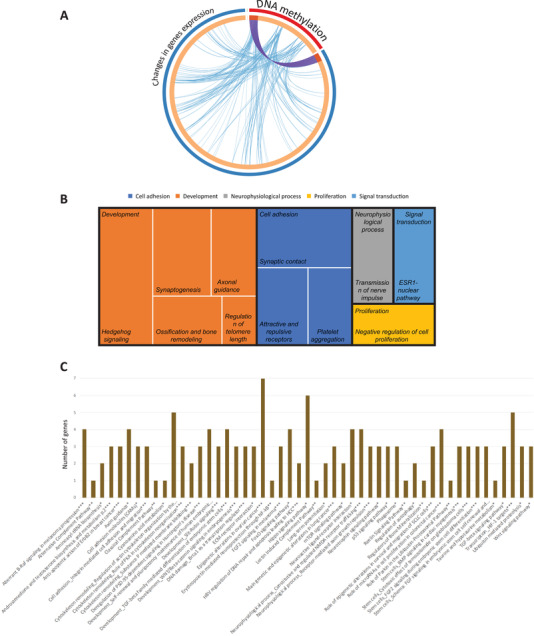
Functional analysis of genes presented epigenetic modifications following CF consumption. A) The overlaps between differentially expressed genes and genes presenting changes at DNA methylation are shown in a circos plot. The circle represents gene lists, purple curves link identical genes, and blue curves link genes that belong to the same enriched ontology term. B) Treemap of gene networks obtained using a text mining approach, C) histology of cellular pathways obtained from BioCarta, KEGG, and Metacore using GeneTrail.

Using in‐silico docking analyses, we first evaluated potential interactions between previously identified major structurally related (−)‐epicatechin metabolites: (−)‐epicatechin‐3′glucuronide and (−)‐epicatechin‐3′sulfate, as well as identified major gut microbiome derived metabolites, 5‐(4′‐hydroxyphenyl)‐γ‐valerolactone‐3′‐sulfate (γVL3′S).^[^ ^33^ ^]^ In‐silico docking analysis has been performed with p65 subunit of NF‐κB transcription factor, that we identified using bioinformatic analysis and which is involved in the regulation of genes identified as correlated with arterial stiffness (such as *CAPNS1*, *RASGRP1*, *PRDX5*, *SLC44A2*) but also in the regulation of genes involved in cell adhesion and inflammatory processes. We also performed docking analysis with JNK, or MAPK10, cell signaling protein involved in signaling pathways that regulate the activity of transcription factors identified, including SP1, JUN, STAT3, SP3, or Myc. We observed that (−)‐epicatechin‐3′glucuronide has a high potential to bind with the p65 subunit of NF‐κB transcription factor with an estimated binding energy of −8.1 kcal mol^−1^ (**Figure** **6**A). Similar to (−)‐epicatechin‐3′glucuronide, (−)‐epicatechin‐4′sulfate can also potentially interact with p65 with a predicted binding energy of −8.8 kcal mol^−1^ (Figure 6B). We also estimated potential interaction of major gut microbiome metabolites with the cell signaling protein JNK and observed that 5‐(4”‐hydrox)‐γ‐valerolactone‐3”‐sulfate had an estimated binding energy of −7.4 kcal mol^−1^ (Figure 6C). This observation suggests that CF metabolites, including gut microbiome derived metabolites, have the potential of binding to cell signaling proteins and transcription factors that could underlie nutrigenomic effects of CFs.

### 3.8 CF Consumption Modulates DNA Methylation Profile

Comparison of genome‐wide DNA methylation profiles before and after CF intervention identified 262 DNA CpG sites with changes (>5%) in the DNA methylation profiles. Analyses of methylation position on chromosomes showed that methylation changes were widespread across the genome. Among the methylation sites, 83 were identified as hypomethylated, that is having decrease in methylation, while 179 were hypermethylated, that is increase in methylation. This observation suggests that regular intake of CF can modify DNA methylation profile of certain genes, with most probes (>68%) presenting an hypermethylation.

**Figure 7:**
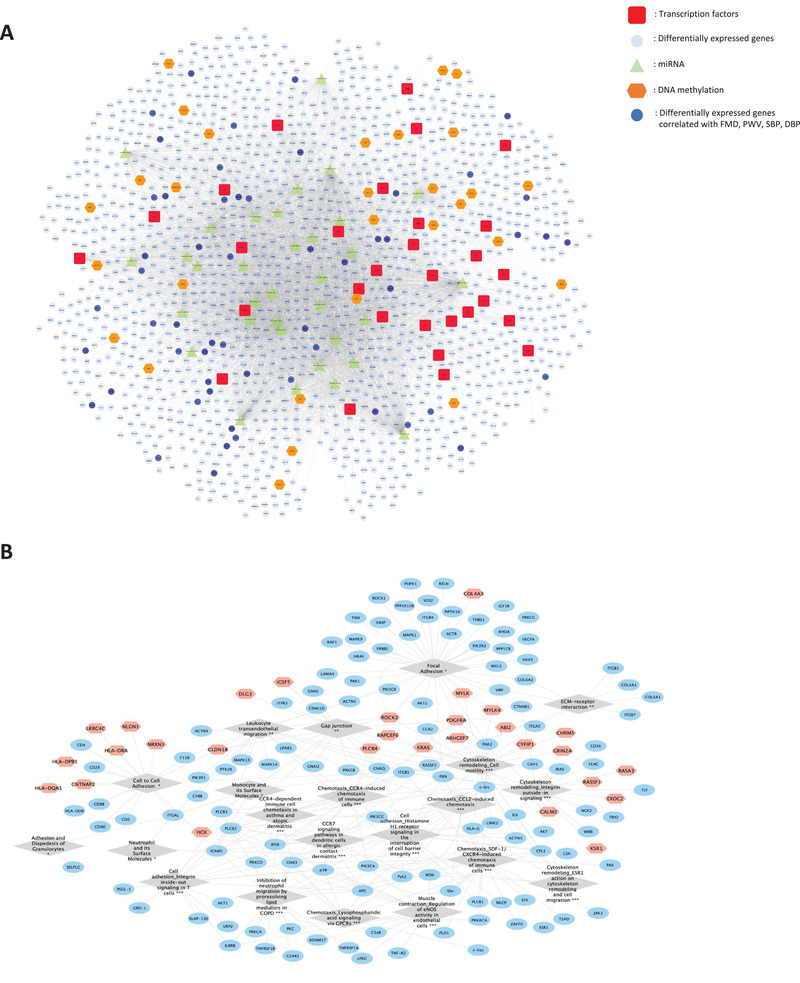
Integration of the multi‐omic data. A) Integrated analyses of differentially expressed genes (blue), genes presentencing changes in DNA methylation (brown), potential transcription factors (red), and potential microRNAs (green). B) Network of genes (blue) presenting changes in expression and DNA methylation (brown) involved in pathways (grey diamond chapped) related to cell adhesion, chemotaxis, and cell mobility.

Analyses of the genome data showed that 173 differentially methylated regions (DMP) were associated with genes, and for other 89 DMPs, we identified nearest genes. Comparison with identified differentially expressed genes, 46 genes were identified in common. This suggests that 10% of genes presenting changes in DNA methylation resulted in observed changes in gene expression as presented in circus plot (**Figure** **7**A). Moreover, the overlaps between differentially expressed genes and genes presenting changes at DNA methylation belonging to the same enriched ontology term have been observed, suggesting that changes in DNA methylation by CF could impact genes regulating similar cellular processes as genes identified as differentially expressed.

Gene network analysis of differentially methylated genes showed that these genes are involved in vascular inflammatory processes including cell adhesion, development or cell signaling (Figure 7B). Similarly, pathway enrichment analysis revealed that the genes having changes in DNA methylation level are implicated in pathways such as those regulating cell adhesion, such as cell adhesion molecules or ECM‐ receptor interaction; cell signaling such as TGFbeta signaling, Wnt, or mTOR signaling pathways, pathways regulating cytoskeleton, for example regulation of actin cytoskeleton organization by the kinase effectors of Rho GTPases or role of PKA in cytoskeleton reorganization, or development (Figure 7C).

### 3.9 Integrative Regulation of Cellular Processes by Modulation of Gene Expression and DNA Methylation

**Figure 8:**
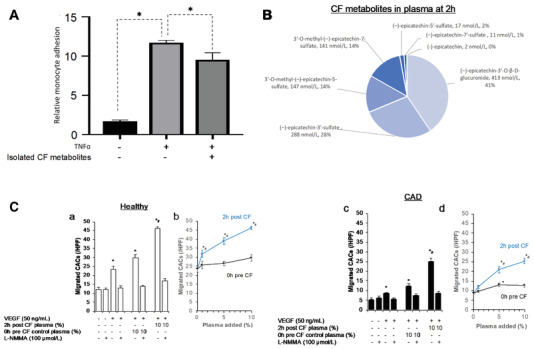
A) Monocytes adhesion after exposure to CF metabolites on endothelial cells in inflammatory condition (*n* = 9; compared to TNFα at 0.1 ng mL^−1^ using ANOVA; * *p<0.05*). B) Profile of CF metabolites in pooled plasma of four healthy volunteers at 2 h after ingestion of 450 mg CF intervention. All metabolites in 0 h pre exposure plasma below limit of detection. C) Effect of post exposure and control plasma on migration of circulating angiogenic cells (CAC) from healthy volunteers (a, b) and patients with coronary artery disease (CAD; c, d) toward a gradient of vascular endothelial growth factor (VEGF) in Boyden chamber over 6 h C) Cell migration significantly differed between healthy and CAD and CF plasma dose‐dependently increased chemotaxis in both groups (Cb+d: repeated measurements ANOVA: *p interaction _interventionxdose_* <0.001,* p interaction _interventionxdosexgroup_ = 0.417*). (Ca+c) The increase in chemotaxis but not chemokinesis was inhibited by the eNOS inhibitor L‐NMMA. (Ca+c) * *p<0.05* versus no additives, # *p<0.05* versus 0h plasma; (Cb+d) * p<0.05 versus 0% plasma, # *p<0.05* versus respective 0h plasma; # *p<0.05* versus 0h plasma.

Although we observe significant overlap in pathways enriched with genes whose expression and/or methylation is changed by CF, similarity becomes rather poor at the gene level. This is suggesting much redundancy in transcriptional versus epigenetic regulation of vascular inflammatory processes. As pathways related to cell chemotaxis, adhesion, and mobility have been identified in common from gene expression changes and changes in DNA methylation, a network around these pathways and associated genes has been constructed (**Figure** **8**A). It shows that by acting on both gene expression and DNA methylation, CF can influence large number of genes involved in networks regulating these processes. Moreover, integrated analyses of differentially expressed genes, genes presenting changes in DNA methylation together with miRNAs as well as identified transcription factors (Figure 8B) showed that these genes are interconnected forming a network of interactions involved in the regulation of identified cellular pathways. This integrated multi‐omics analysis of the data indicates that CF led to DNA methylation changes that may explain some of the gene expression changes but also could be involved in the vascular effects of CF.

### 3.10 Circulating CF Metabolites Decrease Monocyte Adhesion and Maintain Endothelial Integrity via Circulating Angiogenic Cell Migration In vitro

To validate the effects of CF metabolites on monocyte function or monocyte endothelial interaction revealed by bioinformatic analysis of the gene expression and epigenomic data, we performed in vitro experiments. First, an in vitro monocyte adhesion assay was conducted with THP‐1 monocytes and HUVECs to validate the effects of CF metabolites on monocyte adhesion. As expected, induction of inflammatory stress by TNF−α resulted in significant increase in monocyte to endothelial cell adhesion (**Figure** **9**A). CF metabolites were isolated from plasma of volunteers that participated in the CF intervention study and used to pre‐expose cells before induction of stress using TNF−α resulted in a significantly (*p* < 0.05) decreased adhesion of CF metabolites‐treated monocyte to endothelial cells by 14% as compared to control group.

Figure 9Summary of potential interactions between cocoa flavanol (CF) metabolites, cell signaling proteins and transcription factors, impact on expression of genes and correlation with observed phenotypic changes A). Schematic presentation of nutri(epi)genomics changes (presented by a network of mRNA/miRNA/DNA methylation/pathway interactions in the center of the cell), biological processes associated with identified genes in peripheral blood mononuclear cells (PBMC) in volunteers after epicatechin intake and impact on vascular function B).

**Figure:**
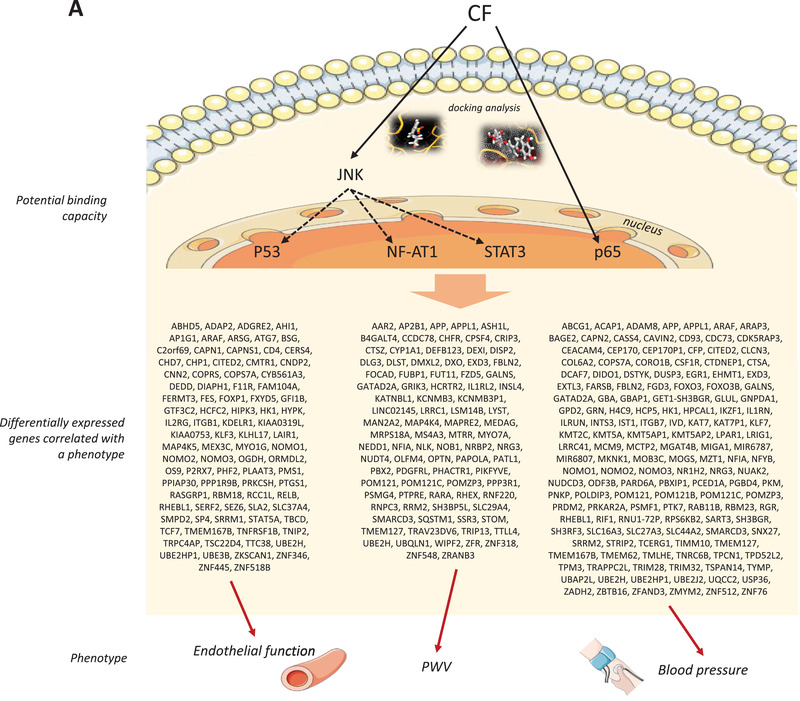

**Figure:**
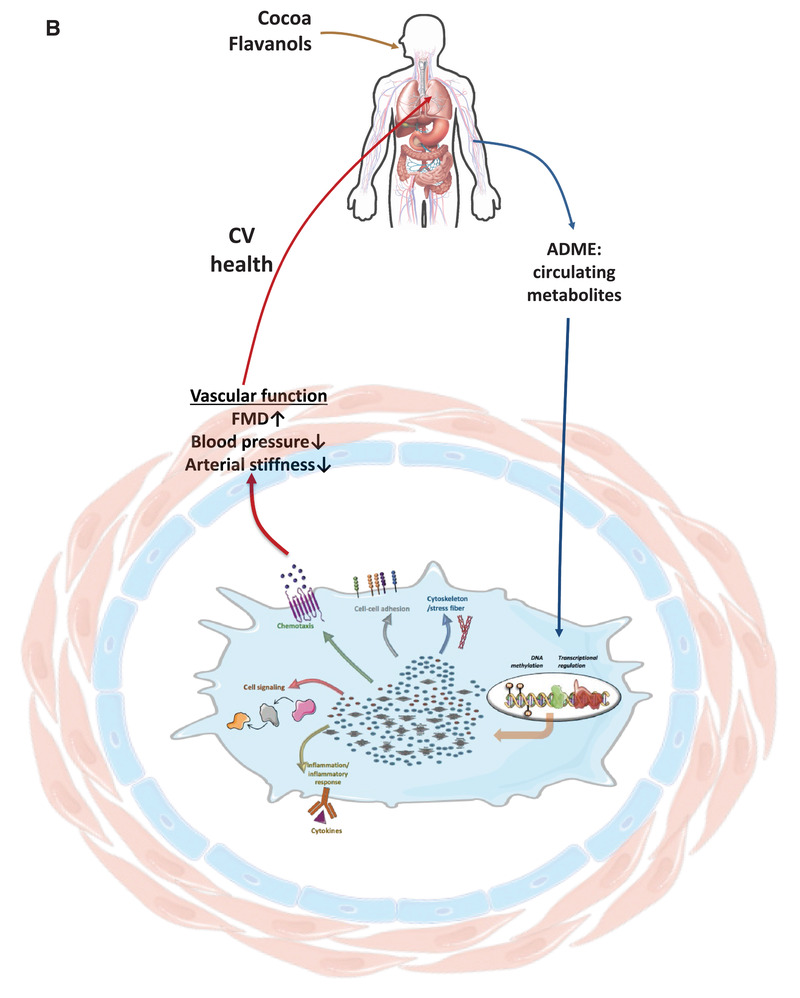

Furthermore, we studied the effect of circulating CF metabolites on chemotaxis of circulating angiogenic cells (CACs) which are part of the mononuclear cells fraction that share monocytic and endothelial characteristics and are important for endothelial maintenance and repair.^[^ ^34^ ^]^ We incubated CACs from five healthy volunteers and five patients with coronary artery disease with plasma taken at 0 h before and at 2 h after CF (450 mg CF, pooled from *n* = 4 healthy males, see composition in Figure 9B). This showed that post CF but not control plasma led to a dose‐dependent increase chemotaxis toward a VEGF gradient of CAC from both CAD patients and healthy volunteers (Figure 9C, *p interaction _dosexintervention_* <0.001, *p interaction _dosexinterventionxgroup_* = 0.417). This response was blocked by the eNOS inhibitor L‐NMMA supporting the important role of eNOS in the mediation of vascular effects. Taken together, the data indicate that CF metabolites improve cellular function as predicted by the integrated multi‐genomic analysis in cells both from healthy and people with cardiovascular disease.

## 4 Discussion

Our clinical study showed that 1 month consumption of CF improved vascular function as reflected by increased in flow‐mediated dilation and decreased systolic and diastolic blood pressure as well as arterial stiffness.^[^ ^2^ ^]^ Our current analysis now shows that the consumption of CF for 1 month elicits significant changes in the gene expression and DNA methylation profile of healthy men which regulate integrity of immune‐endothelial cell barrier functions. Bioinformatic analysis revealed that genes affected by CF consumption are involved in the regulation of inflammation, cell adhesion, and chemotaxis of immune cells. The observed gene expression changes correlated with distinct changes in vascular function, and also inversely correlated with reported gene expression changes seen in CAD. The docking analyses suggested that binding to transcription factors may explain some of the effects, and cell experiments confirmed that CF metabolites enhanced cellular functions as predicted in the gene expression analyses.

Certain genes with changes in the expression following CF consumption presented significant correlations with measured clinical parameters. Ras guanine‐releasing protein 1 (*RasGRP1*) was identified as correlated significantly with changes in FMD. It has been suggested that RasGRP1 is involved in Angiotensin II‐induced periostin expression^[^ ^35^ ^]^ and Ras is one of the signal pathways downstream of VEGF and VEGFR2 signaling,^[^ ^36^ ^]^ which perturbation contributes to endothelial dysfunction assessed by FMD in patients with obstructive sleep apnea.^[^ ^37^ ^]^ Ras signaling pathways, through MAPK signaling pathway can regulate vasocontraction, pathways that can also be regulated by APPL1, gene we identified as corelated with changes with PWV following CF consumption, and APPL1 has been described to be able to prevent age‐ and obesity‐induced impairment in vasodilation and vasoconstriction.^[^ ^38^ ^]^ Interestingly, expression of one gene of MAPK signaling pathway, MAP4K4, was identified as differentially expressed and correlated with PWV. This gene has been described plying a role in the regulation of endothelial injury^[^ ^39^ ^]^ and vascular permeability by reducing focal adhesion. The focal adhesion pathway has been identified as significantly over‐represented in our bioinformatic analysis,^[^ ^40^ ^]^ and is known to be involved in cardiovascular disease development.^[^ ^41^ ^]^

Certain genes with highest changes in the expression following CF consumption presented significant correlations with measured clinical parameters. Change in the expression of *GPR37L1* was correlated with changes in FMD. G protein‐coupled receptors (GPCRs) are a family of seven transmembrane (TM)‐spanning proteins that transmit responses from the extracellular environment by binding to ligands.^[^ ^42^ ^]^ It has been described as involved in the regulation of blood pressure,^[^ ^43^ ^]^ and a genome‐wide association study reported that a SNP near *GPR37L1* gene was associated with sudden cardiac death patients with coronary artery disease (*p* < 0.0001).^[^ ^44^ ^]^ Moreover, gene database analysis showed that this gene is involved in G‐protein cell signaling pathways which regulate focal adhesion, cell cytoskeleton organization and is linked with the MAPK signaling pathway; pathways involved in the regulation of cell adhesion and cell mobility. The expression of this gene is regulated by different transcription factors, such as NFAT1 which activity is regulated by MAPK cell signaling pathways.^[^ ^45^ ^]^ Therefore, we could hypothesis that epicatechin metabolites, by binding to MAPK proteins, as demonstrated, will impact the activity of NFAT1, consequently modulate expression of *GPR37L1*, change in expression of which we identified as correlated with observed changes in FMD (Figure 1A). *ABCB6*, member of adenosine triphosphate (ATP)‐binding cassette (ABC) transporters, was identified as correlated with arterial stiffness, a risk factor linked with vascular ageing and high blood pressure. This gene plays a role in the pathogenesis of vascular diseases, as it is involved in cholesterol homeostasis, regulation of blood pressure, endothelial function, and vascular inflammation, as well as in platelet production and aggregation.^[^ ^46^ ^]^ The expression of this gene is under control of NF‐κB transcription factors. Therefore, our results suggests that CF consumption results in binding of (−)‐epicatechin metabolites to the NF‐κB subunit, as shown by docking analysis, which will result in changes in the NF‐κB activity and consequently modulate expression of ABCB6 genes, which has been correlated with observed changes in arterial stiffness. This could present one of the mechanisms of action of CF underlying their vasculo‐protective effects. A correlation was also observed between changes in the expression of *CD46*, a transmembrane protein, and FMD. It has been reported that this gene present changes in expression in endothelial progenitor cells following a shear‐stress,^[^ ^47^ ^]^ such as the stress induced by blood flow that induces endothelial dysfunction. Interestingly, the expression of this gene is regulated by STAT3 transcription factor which has been identified among the top transcription factors involved in the gene expression modifications following CF consumption in our study. The activity of STAT3 can be regulated by MAPK cell signaling pathways, for which we observed potential binding with (−)‐epicatechin metabolites. Changes in the expression of *LGR6* (Leucine Rich Repeat Containing G Protein‐Coupled Receptor 6) showed correlation with changes in three clinical parameters: central systolic blood pressure, total cholesterol, and LDL cholesterol. Its function is not well known but the protein coded by this gene is involved in Wnt signaling pathway, a pathway that has been described to be involved in the regulation of blood pressure^[^ ^48^ ^]^ but also in cholesterol metabolism.^[^ ^49^ ^]^ The expression of this gene is regulated by different transcription factors including TP53, WT1, STAT1, or MYC, transcription factors which have been identified using our bioinformatic analysis. Among these transcription factors, the activity of TP53 can be regulated by the JNK MAP kinase cell signaling protein, suggesting that (−)‐epicatechin metabolites, by interacting with JNK, can modulate the activity of TP53 which results in changes in the expression of *LGR6*. This protein is correlated with changes in total and LDL cholesterol as well as FMD (Figure 1A). Therefore, the modulation of the expression of these genes by flavanols can play, directly or indirectly, a potentially important role in the vasculo‐protective effect of these food bioactives.

During inflammation, proinflammatory cytokines induce the production of chemokines that then attract leukocytes to the site of inflammation. Controlled leukocyte recruitment is crucial for the generation of an immune response, but inappropriate trafficking can lead to the development of chronic inflammatory diseases.^[^ ^50^ ^]^ Chemokines and chemokine receptors have been implicated in development of atherosclerosis; at the initiation phase of plaque formation during leukocyte adhesion and chemotaxis and during progression, but also in development of cardiovascular diseases.^[^ ^51^ ^]^ Among the CF responsive genes, we observed a significant decrease in expression of chemokine CC motif ligand 4 (CCL4) which may elicit cardioprotective effects by reducing vascular inflammation. Recent findings indeed demonstrate that CCL4, and its receptor CCR5, play diverse roles in the inflammatory events underlying cardiovascular diseases and is upregulated in atherosclerosis and myocardial infarction, enhancing adhesion molecule expression and accelerating the vascular inflammation response.^[^ ^52^ ^]^ Moreover, our study also showed a decrease in expression of the chemokine *CXCL4*. Pre‐clinical and clinical studies revealed the participation of CXCL4 and its receptor, CXCR3, in multiple cardiovascular diseases of different etiologies including atherosclerosis, hypertension, cardiac hypertrophy, and heart failure.^[^ ^53^ ^]^ In addition, the recently reported association of COVID‐19 vascular pathogenesis with elevated expression of a number of cytokines, including CCL4 and CXCL family chemokines,^[^ ^54^ ^]^ suggests that CF may also protect against vascular inflammatory damage induced by the virus.

Following chemotaxis of immune cells to damaged sites of blood vessels, infarcted heart or other organs, extensive transendothelial migration of leukocytes to subendothelial locations^[^ ^51^ ^]^ can evolve into cardiovascular pathologies such as atherosclerosis, myocardial infarction, stroke, and ischemia–reperfusion injury.^[^ ^55^ ^]^ Migration of immune cells through the vessel wall requires intracellular signaling and cellular actin cytoskeleton and actomyosin modifications. Actomyosin contraction is dependent on myosin light chain phosphatases that can inhibit stress‐fiber polymerization. In our study, we observed a significant increase in expression of genes coding these protein phosphatases, such as *PPP1* and *PPP2* regulatory subunits. In the cells, F‐actin polymerization is also regulated by focal adhesion signaling pathway and GTPase‐activating proteins. In our study, over 50 genes involved in focal adhesion, Ras/Rap1 signaling pathway have been identified as having expression modulated by CF. Among them is Ras GTPase‐Activating Protein 3 (RASA3) which expression was increased by CF. It has been suggested that deletion or knockdown of *Rasa3* is associated with hyperactivation of the small GTPase Rap1 and, therefore, F‐actin polymerization.^[^ ^56^ ^]^ Increased expression of this gene in our study is suggestive of lower cytoskeleton polymerization and therefore decreased immune cell infiltration into vascular wall, which contributes in prevention of atherosclerosis and vascular dysfunctions. Besides benefits in cardioprotection, CF could also lower accumulation of immune cells in the lung during COVID‐19 infection and decrease the severity of the disease as in COVID‐19 patients, migration and infiltration of immune cells to the site of infection, particularly in lung tissues, of COVID‐19 patients was particularly observed.^[^ ^57^ ^]^

In circulation, hypoxia causes rapid systemic vasodilation, and it has been suggested that hypoxia is mediated by the HIF hydroxylase system which plays an interface with processes, such as cardiovascular development, angiogenesis, endothelial function or vasomotor regulators.^[^ ^58^ ^]^ Inactivation of HIF‐1α results in reduced expression of nitric oxide synthase 2 which is associated with increased systemic blood pressure.^[^ ^59^ ^]^ A factor inhibiting Hypoxia‐inducible factor 1‐alpha protein (HIF‐1), Hypoxia Inducible Factor 1 Subunit Alpha Inhibitor (FIH‐1), binds to HIF‐1α and inhibits its transactivation.^[^ ^60^ ^]^ We observed that CF consumption decreases (–1.2) the expression of FIH‐1, profile that suggest increase in HIF1 activity and potentially lower blood pressure as we observed in our clinical trial.

Bioinformatic analyses of gene expression and epigenetic modifications induced by CF consumption also revealed genes involved in inflammation and lipid metabolism. IL‐10 is a well‐known anti‐inflammatory cytokine, known to modulate lipid metabolism, promote M2 macrophage differentiation,^[^ ^61^ ^]^ inhibition of matrix metallo‐proteases^[^ ^62^ ^]^ and provides protection against atherosclerosis. Weak upregulation of IL‐10 receptor A and B (*IL10RA/B*) supporting IL10 signaling could be observed upon cocoa‐flavanol intake with fold changes of 1.15 and 1.11 respectively. With respect to downregulated genes, cathepsin G (*CTSG*) showed decreased expression after cocoa‐flavanol uptake. Cathepsin G is a neutrophil serine protease that is able to activate the proinflammatory cytokines TNF‐α and IL‐1β, extracellular matrix remodeling and to reduce cholesterol efflux and lipid metabolism.^[^ ^63^ ^]^ Taken together, the gene expression profile is suggestive of an anti‐inflammatory effect of regular consumption of CF therefore presenting potential preventive properties regarding vascular related diseases but also inflammatory related diseases, such as COVID‐19. An upregulation in expression was also observed in the ABC‐binding cassette transporter family genes, *ABCA1* (1.14) and *ABCG1* (FC 1.27), which enhance cholesterol efflux from foam cells.^[^ ^64^ ^]^ Also, the low‐density lipoprotein receptor‐related protein 1 (*LRP1*) gene was downregulated after CF consumption, with a fold change of 1.22. LRP1 is multi ligand transmembrane receptor that binds to low‐density lipoproteins (LDL) and is involved in lipid metabolism, cellular migration, and immune response.^[^ ^65^ ^]^ Reduced LRP1 expression through diet has already been reported in a study by Konstantinidou et al.^[^ ^66^ ^]^ after intake of olive oil, further supporting the anti‐inflammatory capacity of CF.

Only few studies have addressed nutri(epi)genomic changes in gene expression and DNA methylation involved in the cardioprotective effects of CF consumption. One study has identified 87 differentially expressed genes in PBMCs of vascular arterial disease patients, genes involved in mediating processes like immune response, inflammation, apoptosis, cell signaling, or platelet activation and aggregation.^[^ ^67^ ^]^ Health benefits of CF consumption may attributed to reversing adverse gene expression associated with vascular arterial disease. For example, *B3GNT2* (UDP‐GlcNAc:BetaGal Beta‐1,3‐N‐Acetylglucosaminyltransferase 2) has been identified as up‐regulated in patients with arterial disease while CF significantly down‐regulated its expression. *DNAJB6*, member of DnaJ Heat Shock Protein Family, was also identified as up‐regulated in patients with vascular disease, while CF decreased its expression, as similar for transforming growth factor, beta receptors (*TGFBR*). Changes in the expression of 744 genes in PBMCs have also been identified in patients with hypertension.^[^ ^68^ ^]^ Genes that have been identified potentially associated with hypertension are involved in processes regulating immune response, chemotaxis, inflammation or cell signaling, functions that have been also identified as affected by CF. Among the genes in common with our study are *NME4*, *IGF1R*, or *PIK3C*, suggesting again that CF by modulating the expression of genes can decrease blood pressure in humans. Furthermore, our gene expression study showed that CF regulate numerous genes involved in regulation of their adhesion to and transmigration through vascular endothelial, a profile of which is suggestive of lower adhesion and transmigration. Immune cells can also contribute to arterial dysfunction, arterial stiffness, and impaired endothelium‐dependent dilation by infiltration into aorta and mesenteric vascular arcade, processes which also increase with aging.^[^ ^69^ ^]^ Our data showed that post CF exposure plasma stimulates eNOS dependent chemotaxis in CACs, which exhibit phenotypic similarities with monocytes and endothelial cells. Similarly, lifestyle interventions, such as caloric restriction, also improve arterial function by normalizing age‐related arterial immune cell infiltration.^[^ ^69^ ^]^ Improvement in these functions by CF via modulation of gene expression and DNA methylation could result in prevention of development of cardiovascular diseases. Observed negative correlation between gene expression profile obtained following CF consumption and gene expression profile of CAD patients suggests that consumption of this bioactive present cardioprotective properties.

Use of bioinformatic tools allowed us to identify multiple flavanol responsive microRNAs (miRNAs), targeting various mRNAs involved in vascular disease.^[^ ^15^, ^70^, ^71^ ^]^ Changes in mir‐16‐5p expression have been associated with coronary artery disease^[^ ^72^ ^]^ and in hypertensive heart disease.^[^ ^73^ ^]^ Besides this miRNA, mir‐26b‐5p was identified as playing a role in the pathogenesis of hypertension in hypertensive patients with left ventricular hypertrophy^[^ ^74^ ^]^; mir‐335‐5p can target eNOS a key player in the endothelial regulation of vascular reactivity and blood pressure^[^ ^75^ ^]^; mir‐92a‐3p expression is associated with 24‐h mean systolic blood pressure in hypertensive patients^[^ ^76^ ^]^; mir‐124‐3p participates in the regulation of vascular reactivity after hypoxia.^[^ ^77^ ^]^ Interestingly, it has been already observed that polyphenols can reverse the expression of these CVD associated miRNAs. For example, flavanols, quercetin, and quercetin‐glucoside, have been shown to change the expression of mir‐16‐5p^[^ ^78^, ^79^ ^]^ in in vitro studies; the expression of mir‐26b‐5p was changed after consumption of polyphenol‐enriched virgin olive oil in healthy volunteers^[^ ^80^ ^]^; while mir‐335 expression changes were found after consumption of polyphenol‐rich tea in mice model of obesity.^[^ ^81^ ^]^ Similarly, other bioinformatic studies identified mir‐335‐5p, let‐7b‐5p, mir‐26b‐5p, or mir‐16‐5p as flavanol sensitive targets.

Each type of omics data, on its own, classically provides a list of differences between two studied conditions and can give insight as to which biological pathways or processes are different between the disease and control groups. However, analysis of only one data type of omics data is limited and does not provide detailed molecular mechanisms. To study complex biological processes holistically, it is crucial to take an integrative approach that combines multi‐omics data to highlight the interrelationships of the involved biomolecules and their functions, which can provide a deeper understanding of the processes and dynamic interactions involved in human diseases or treatment.^[^ ^82^ ^]^ However, this integrated multiomics approach has been little applied in nutrition research. Our study is one of the first to use such holistic approach to decipher as precisely as possible molecular mechanisms of action of polyphenols in humans. Using such approach, we demonstrated that CF can regulate simultaneously genes at different levels which form a network of interactions, and together regulate different cellular processes which can be associated with their health properties (Figure 1B). We showed that CF can change DNA methylation and expression of genes which can be affected by changes in DNA methylation but also through transcription factor activity and miRNAs. This provide a most comprehensive study of molecular mechanisms of this bioactive and reveal regulators involved at the molecular level.

However, in our present study, a major limitation is that we were not able to obtain endothelial cell samples from the volunteers and therefore cannot assess the direct effects of CF metabolites on vasculature. The similarities of CAC with endothelial cells however indicate that the biological processes affected may not be restricted to mononuclear cells, but extend to other cells like endothelial cells. Another point worth noting is that the theobromine content of the interventions was lower than other studies.^[^ ^83^ ^]^ We and others have previously shown that methylxanthines can significantly increase the biological activity of CF in a dose‐dependent fashion with an ED_50_ at 80 mg^[^ ^84^ ^]^ which is close to the daily amount provided in the present study. This indicates that one would expect greater effects if CF products with higher theobromine content were consumed. Another limitation is that we analyzed changes in expression of genes only in male volunteers to exclude the influence of the oestrogen cycles in female volunteers. We have previously shown with another polyphenol, curcumin, that large sex differences can exist in genomic responsiveness.^[^ ^85^ ^]^

## 5 Conclusion

In conclusion, our study provides evidence that CF consumption can modulate important gene networks in whole blood cells that are involved in interactions with the endothelium. Because of multi‐omic modes of action, our data suggest that there are no individual genes/proteins involved in the health properties of this bioactive but rather a multi‐target mode of action. This study identified groups of genes and major functional cell processes modulated by consumption of CF in humans. Future studies therefore need to consider this mode of action, use multi‐omic approach and go into deeper analysis including genomic, epigenomic and transcription factor studies.

## Conflict of Interest

The authors declare no conflict of interest.

## Supporting information

Supporting Information

## Data Availability

Research data are not shared.
