# Supplementary material for: Flavanol Consumption in Healthy Men Preserves Integrity of Immunological‐Endothelial Barrier Cell Functions: Nutri(epi)genomic Analysis
Source: Mol Nutr Food Res. 2022 Feb 16;66(21):2100991. doi: 10.1002/mnfr.202100991 (PMC9787825; doi:10.1002/mnfr.202100991)
Supplement: Supplementary file 1 — Supporting Information [file MNFR-66-2100991-s001.pptx]

## Slide 1
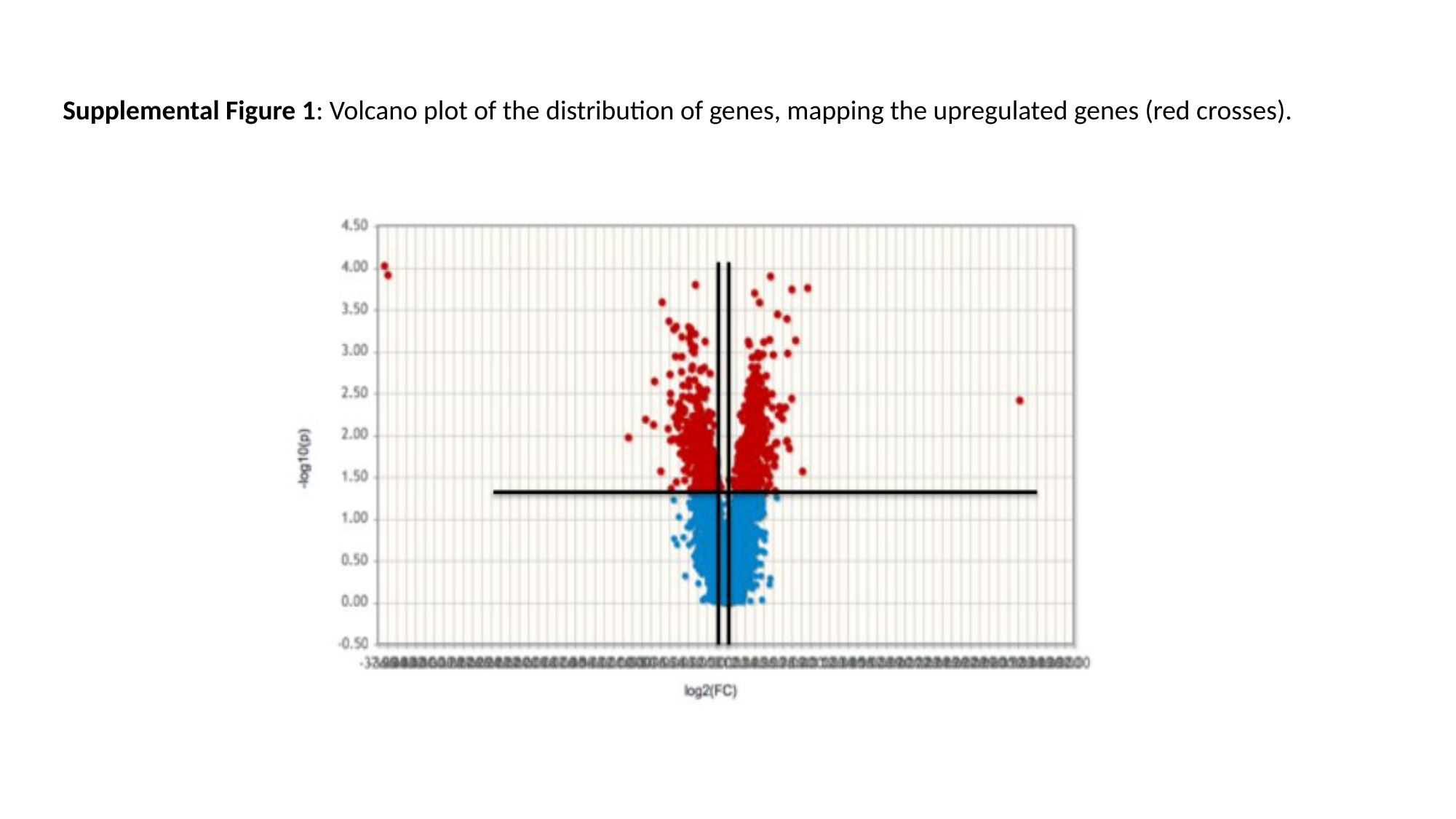

Supplemental Figure 1: Volcano plot of the distribution of genes, mapping the upregulated genes (red crosses).

## Slide 2
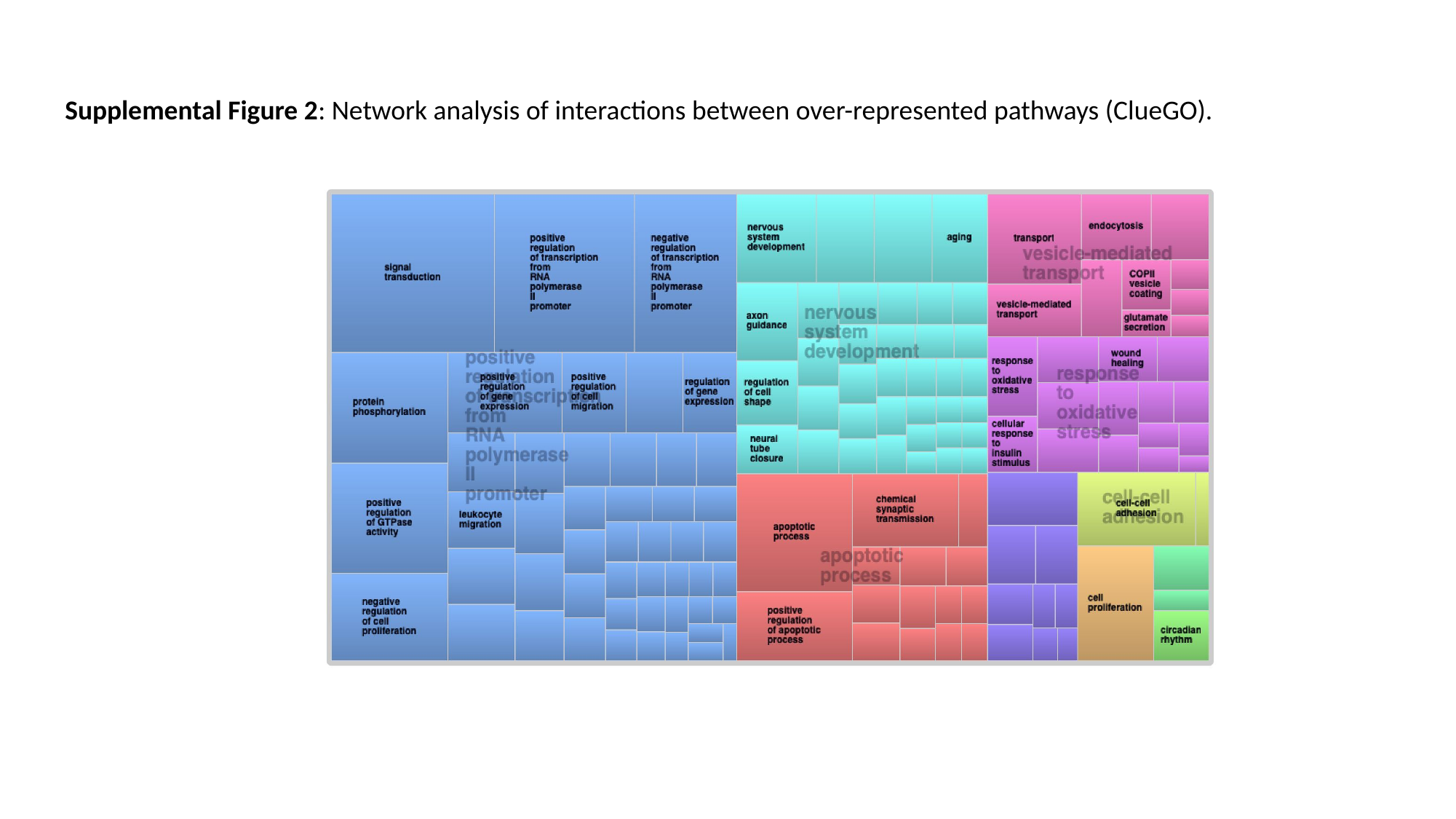

Supplemental Figure 2: Network analysis of interactions between over-represented pathways (ClueGO).

## Slide 3
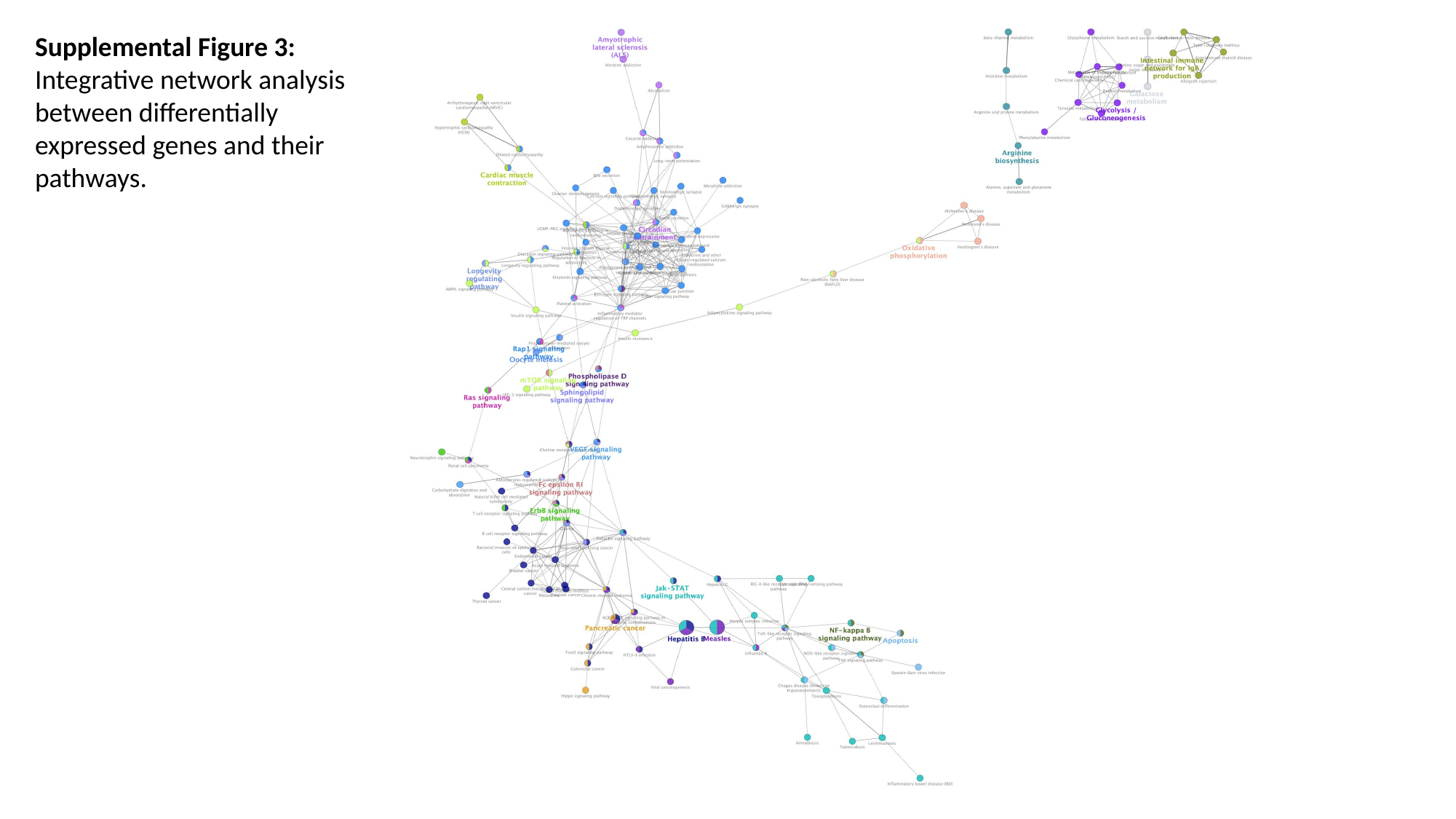

Supplemental Figure 3: Integrative network analysis between differentially expressed genes and their pathways.

## Slide 4
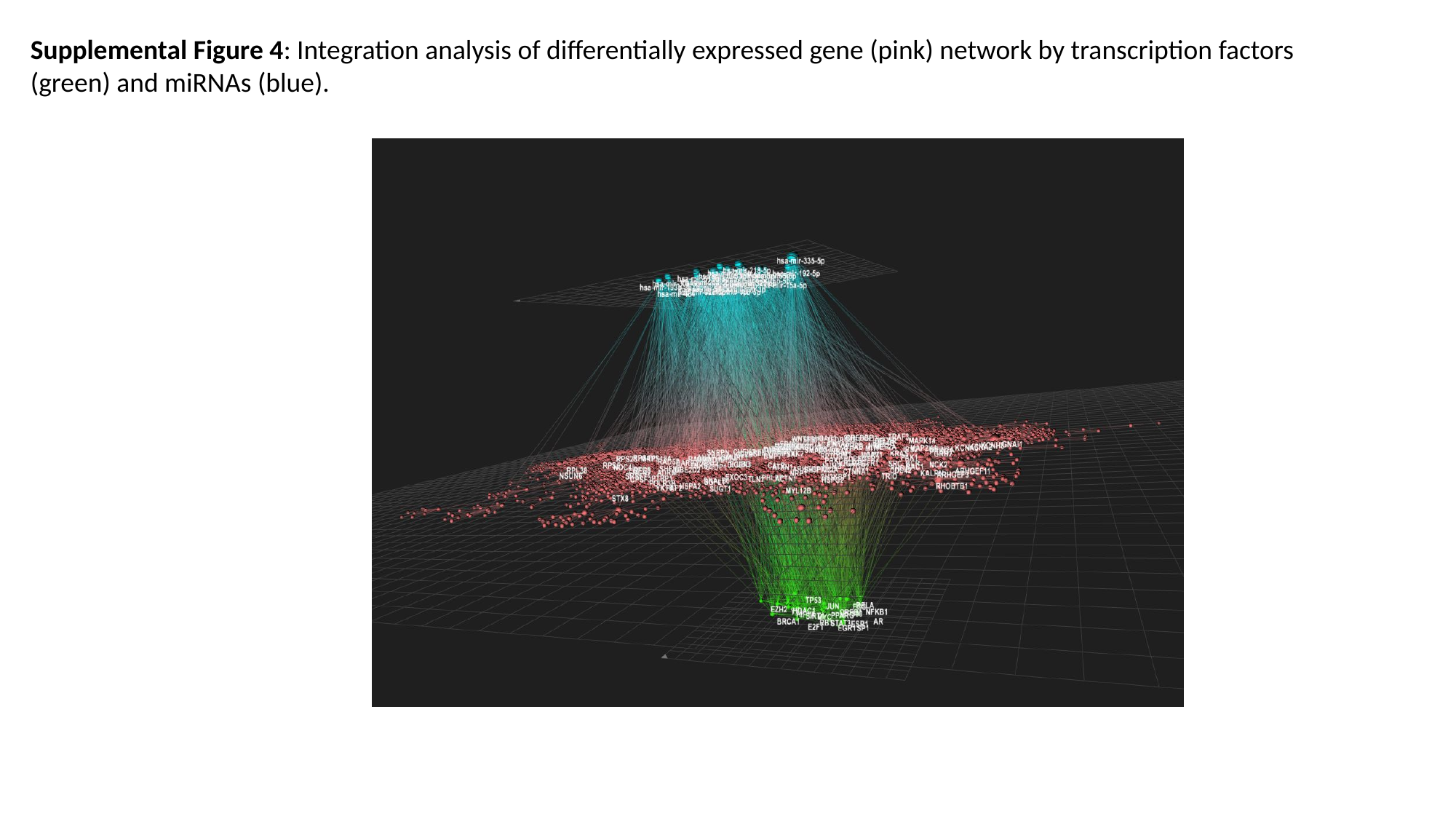

Supplemental Figure 4: Integration analysis of differentially expressed gene (pink) network by transcription factors (green) and miRNAs (blue).

## Slide 5
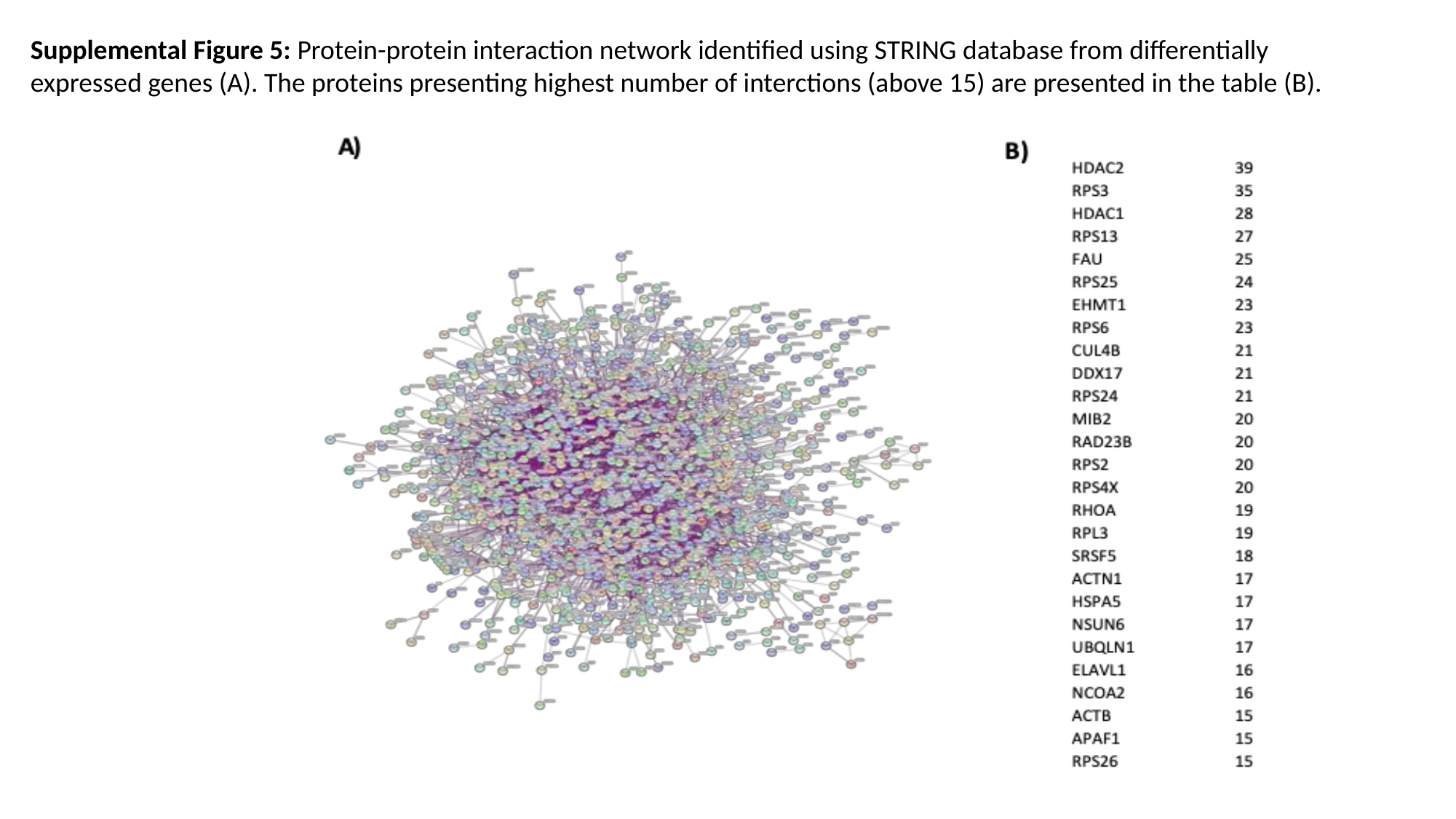

Supplemental Figure 5: Protein-protein interaction network identified using STRING database from differentially expressed genes (A). The proteins presenting highest number of interctions (above 15) are presented in the table (B).
